# Supplementary figures and images for: KEAP1 retention in phase-separated p62 bodies drives liver damage under autophagy-deficient conditions (part 1 of 2)
Source: EMBO Rep. 2025 May 28;26(13):3384–410. doi: 10.1038/s44319-025-00483-9 (PMC12238652; doi:10.1038/s44319-025-00483-9)

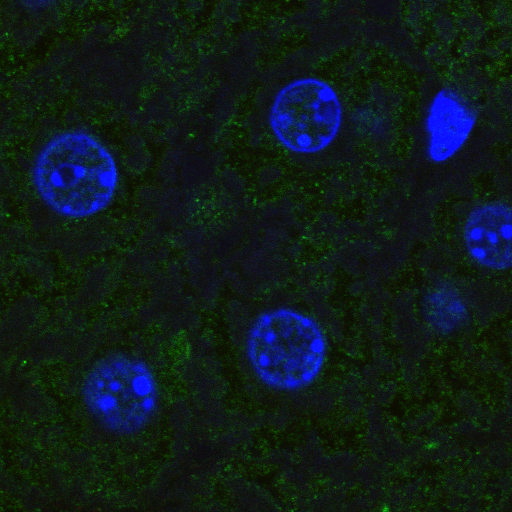

Supplement: Supplementary file 2 — Source data Fig. 1 [file 44319_2025_483_MOESM2_ESM.zip › Fig. 1 source data/Fig. 1A/Fig1A_Atg7FF p62S351A_merge.tif]

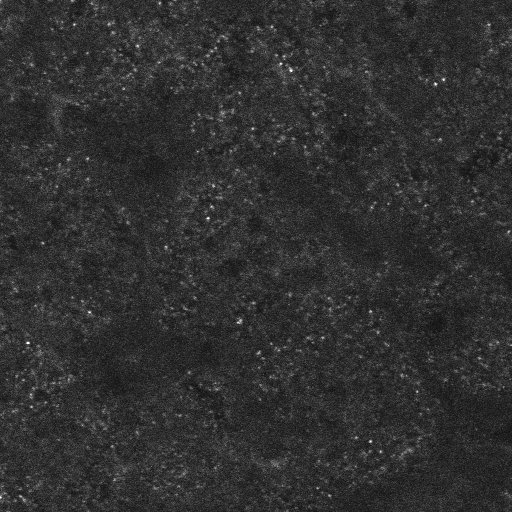

Supplement: Supplementary file 2 — Source data Fig. 1 [file 44319_2025_483_MOESM2_ESM.zip › Fig. 1 source data/Fig. 1A/Fig1A_Atg7FF_KEAP1.tif]

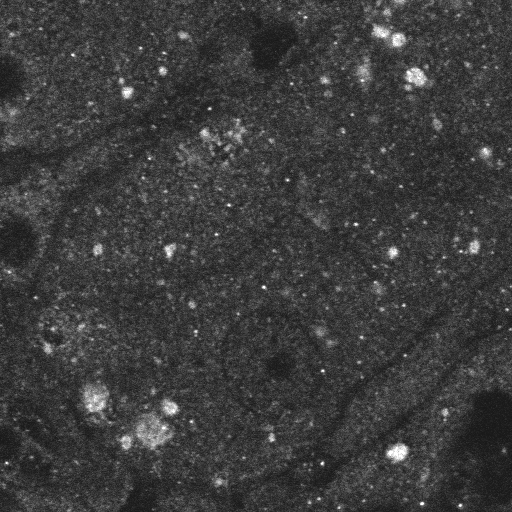

Supplement: Supplementary file 2 — Source data Fig. 1 [file 44319_2025_483_MOESM2_ESM.zip › Fig. 1 source data/Fig. 1A/Fig1A_Atg7FF AlbCre_KEAP1.tif]

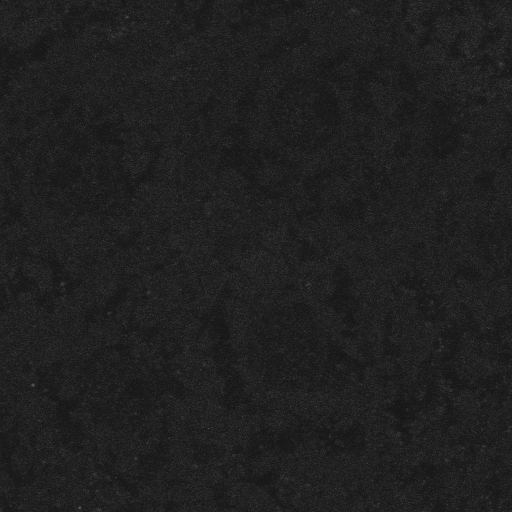

Supplement: Supplementary file 2 — Source data Fig. 1 [file 44319_2025_483_MOESM2_ESM.zip › Fig. 1 source data/Fig. 1A/Fig1A_Atg7FF p62S351A_p62.tif]

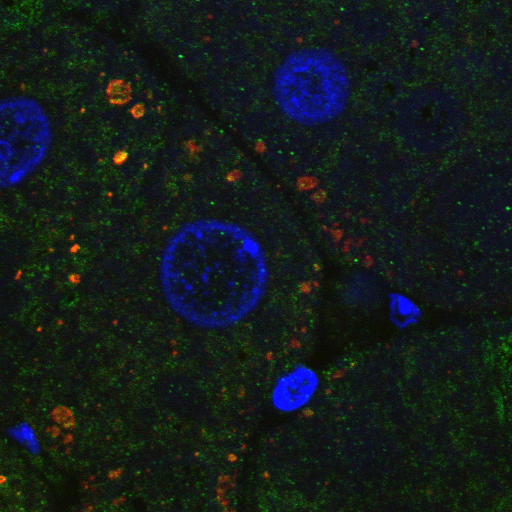

Supplement: Supplementary file 2 — Source data Fig. 1 [file 44319_2025_483_MOESM2_ESM.zip › Fig. 1 source data/Fig. 1A/Fig1A_Atg7FF AlbCre p62S351A_merge.tif]

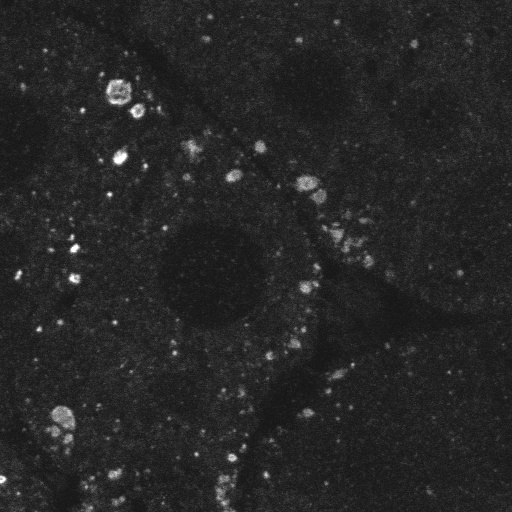

Supplement: Supplementary file 2 — Source data Fig. 1 [file 44319_2025_483_MOESM2_ESM.zip › Fig. 1 source data/Fig. 1A/Fig1A_Atg7FF AlbCre p62S351A_p62.tif]

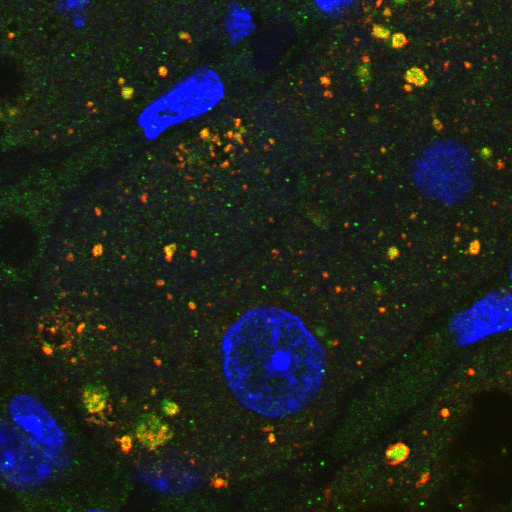

Supplement: Supplementary file 2 — Source data Fig. 1 [file 44319_2025_483_MOESM2_ESM.zip › Fig. 1 source data/Fig. 1A/Fig1A_Atg7FF AlbCre_merge.tif]

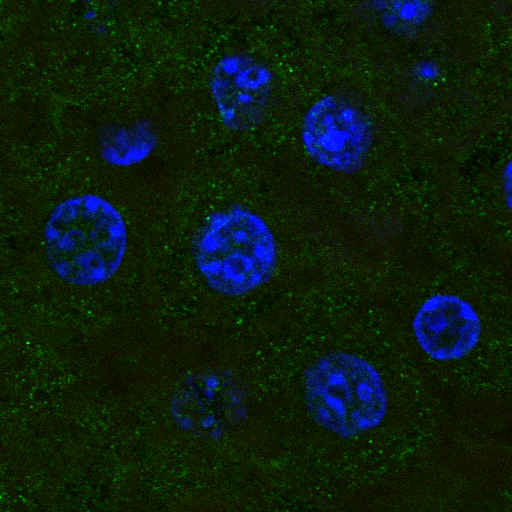

Supplement: Supplementary file 2 — Source data Fig. 1 [file 44319_2025_483_MOESM2_ESM.zip › Fig. 1 source data/Fig. 1A/Fig1A_Atg7FF_merge.tif]

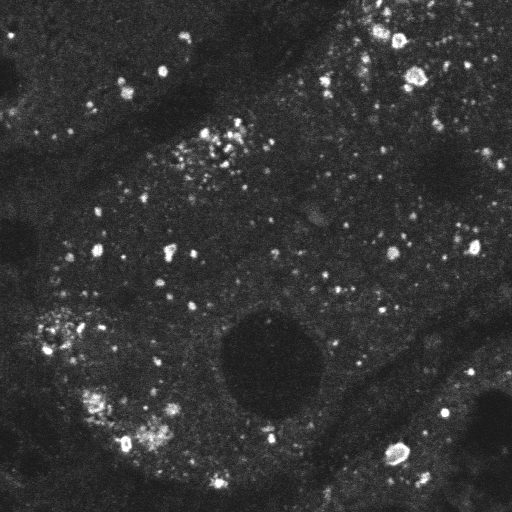

Supplement: Supplementary file 2 — Source data Fig. 1 [file 44319_2025_483_MOESM2_ESM.zip › Fig. 1 source data/Fig. 1A/Fig1A_Atg7FF AlbCre_p62.tif]

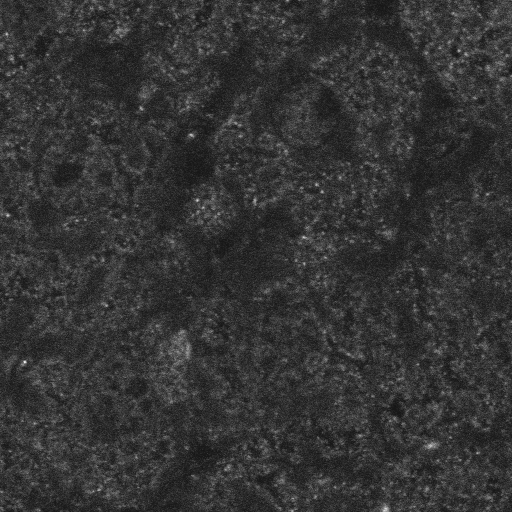

Supplement: Supplementary file 2 — Source data Fig. 1 [file 44319_2025_483_MOESM2_ESM.zip › Fig. 1 source data/Fig. 1A/Fig1A_Atg7FF p62S351A_KEAP1.tif]

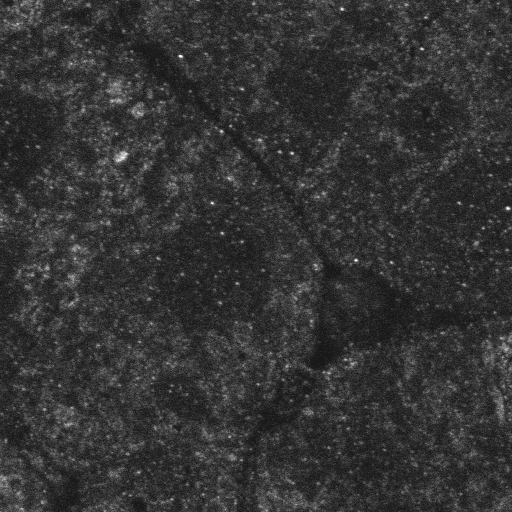

Supplement: Supplementary file 2 — Source data Fig. 1 [file 44319_2025_483_MOESM2_ESM.zip › Fig. 1 source data/Fig. 1A/Fig1A_Atg7FF AlbCre p62S351A_KEAP1.tif]

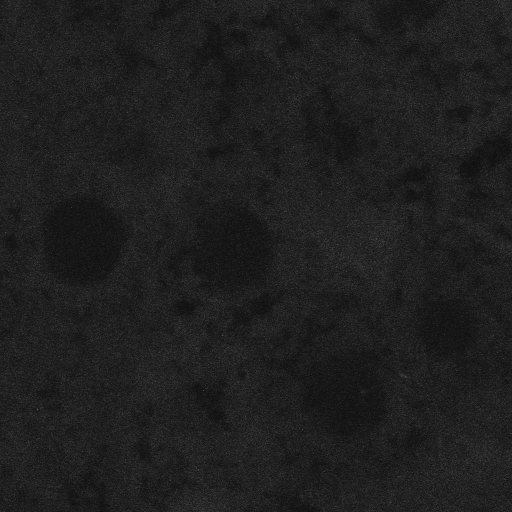

Supplement: Supplementary file 2 — Source data Fig. 1 [file 44319_2025_483_MOESM2_ESM.zip › Fig. 1 source data/Fig. 1A/Fig1A_Atg7FF_p62.tif]

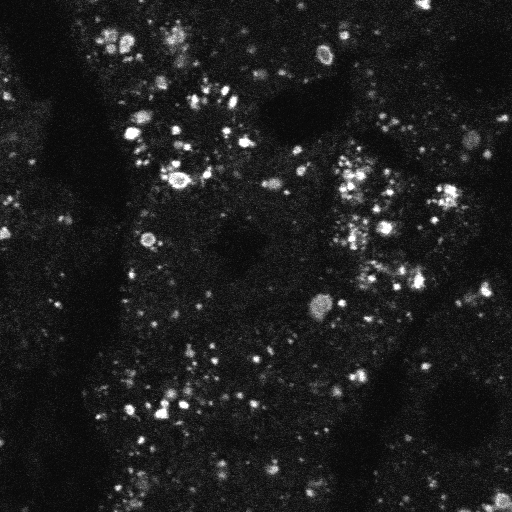

Supplement: Supplementary file 2 — Source data Fig. 1 [file 44319_2025_483_MOESM2_ESM.zip › Fig. 1 source data/Fig. 1B/Fig1B_Atg7FF AlbCre_p62.tif]

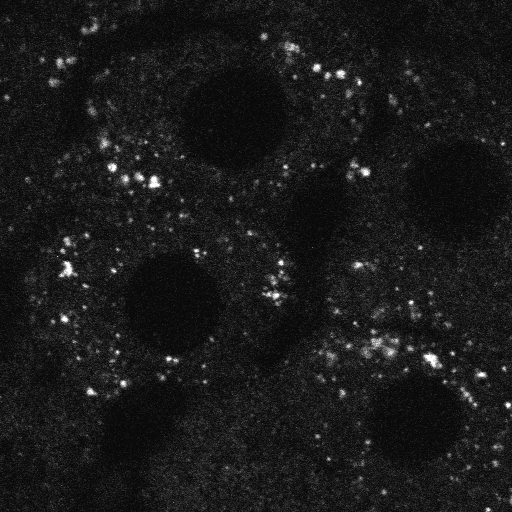

Supplement: Supplementary file 2 — Source data Fig. 1 [file 44319_2025_483_MOESM2_ESM.zip › Fig. 1 source data/Fig. 1B/Fig1B_Atg7FF AlbCre p62T352A_p62.tif]

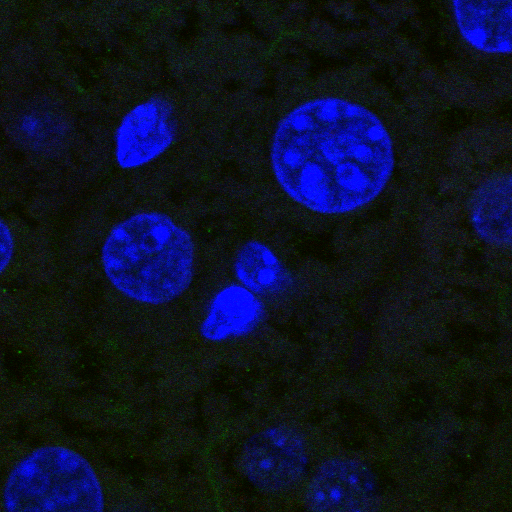

Supplement: Supplementary file 2 — Source data Fig. 1 [file 44319_2025_483_MOESM2_ESM.zip › Fig. 1 source data/Fig. 1B/Fig1B_Atg7FF_merge.tif]

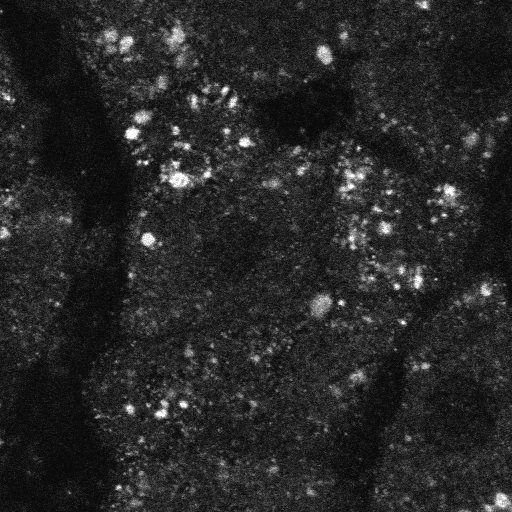

Supplement: Supplementary file 2 — Source data Fig. 1 [file 44319_2025_483_MOESM2_ESM.zip › Fig. 1 source data/Fig. 1B/Fig1B_Atg7FF AlbCre_KEAP1.tif]

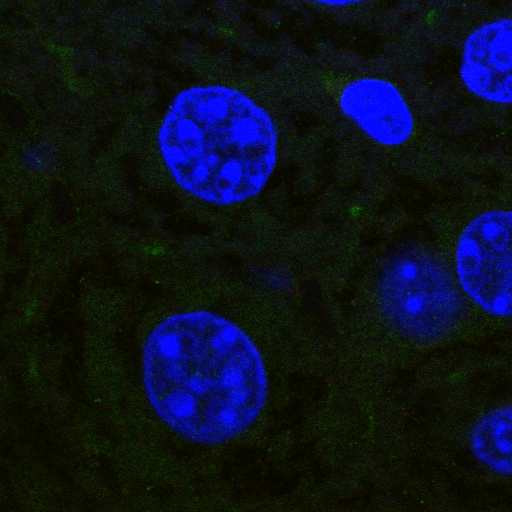

Supplement: Supplementary file 2 — Source data Fig. 1 [file 44319_2025_483_MOESM2_ESM.zip › Fig. 1 source data/Fig. 1B/Fig1B_Atg7FF p62T352A_merge.tif]

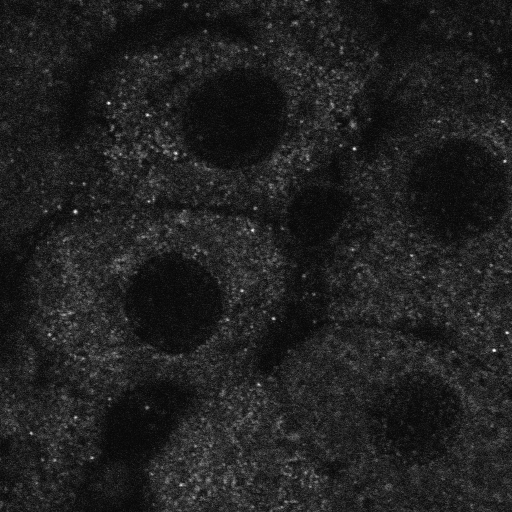

Supplement: Supplementary file 2 — Source data Fig. 1 [file 44319_2025_483_MOESM2_ESM.zip › Fig. 1 source data/Fig. 1B/Fig1B_Atg7FF AlbCre p62T352A_KEAP1.tif]

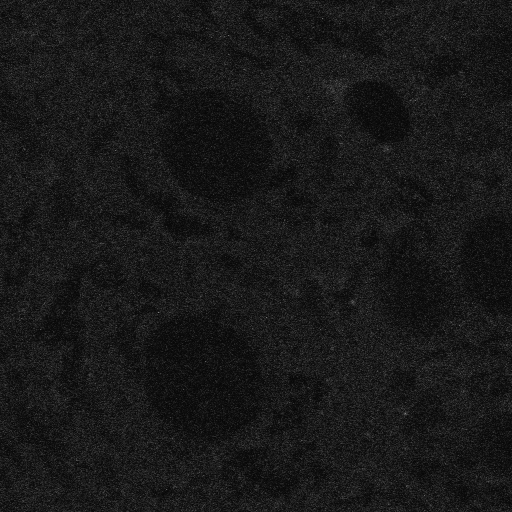

Supplement: Supplementary file 2 — Source data Fig. 1 [file 44319_2025_483_MOESM2_ESM.zip › Fig. 1 source data/Fig. 1B/Fig1B_Atg7FF p62T352A_p62.tif]

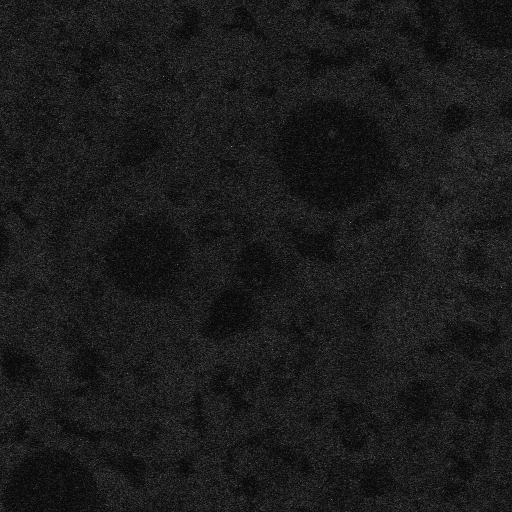

Supplement: Supplementary file 2 — Source data Fig. 1 [file 44319_2025_483_MOESM2_ESM.zip › Fig. 1 source data/Fig. 1B/Fig1B_Atg7FF_p62.tif]

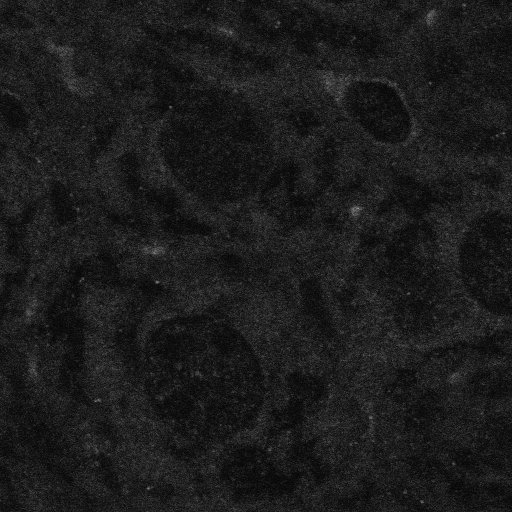

Supplement: Supplementary file 2 — Source data Fig. 1 [file 44319_2025_483_MOESM2_ESM.zip › Fig. 1 source data/Fig. 1B/Fig1B_Atg7FF p62T352A_KEAP1.tif]

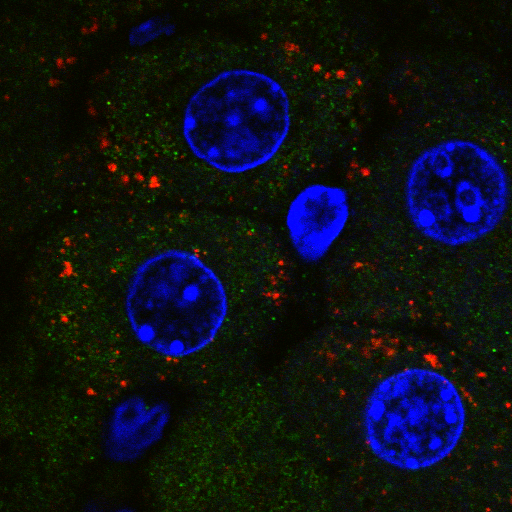

Supplement: Supplementary file 2 — Source data Fig. 1 [file 44319_2025_483_MOESM2_ESM.zip › Fig. 1 source data/Fig. 1B/Fig1B_Atg7FF AlbCre p62T352A_merge.tif]

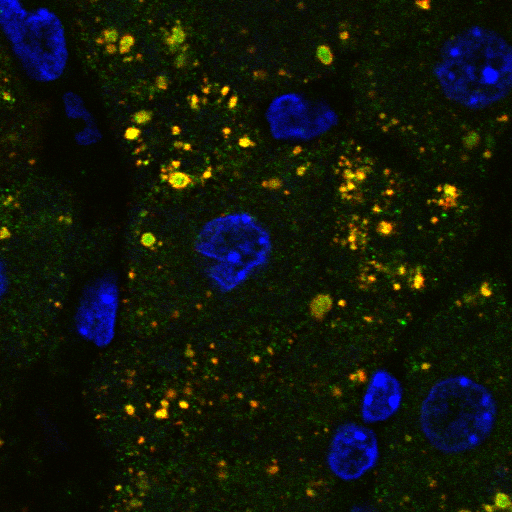

Supplement: Supplementary file 2 — Source data Fig. 1 [file 44319_2025_483_MOESM2_ESM.zip › Fig. 1 source data/Fig. 1B/Fig1B_Atg7FF AlbCre_merge.tif]

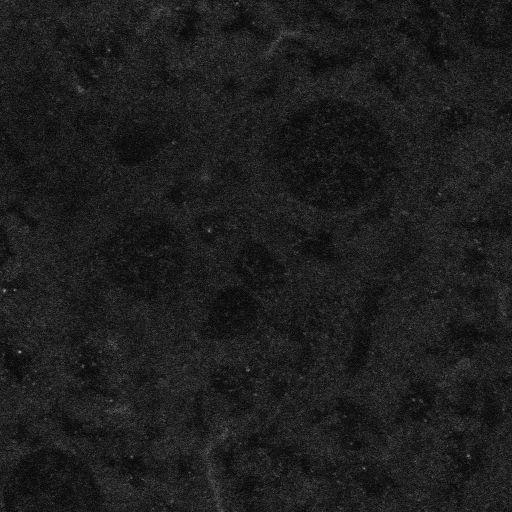

Supplement: Supplementary file 2 — Source data Fig. 1 [file 44319_2025_483_MOESM2_ESM.zip › Fig. 1 source data/Fig. 1B/Fig1B_Atg7FF_KEAP1.tif]

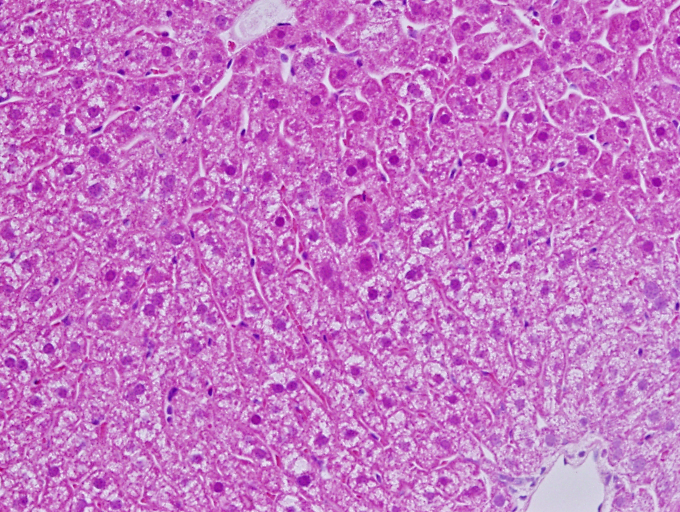

Supplement: Supplementary file 7 — Source data Fig. 6 [file 44319_2025_483_MOESM7_ESM.zip › Fig. 6 source data/Fig. 6C /Fig6C_cont_upr.tif]

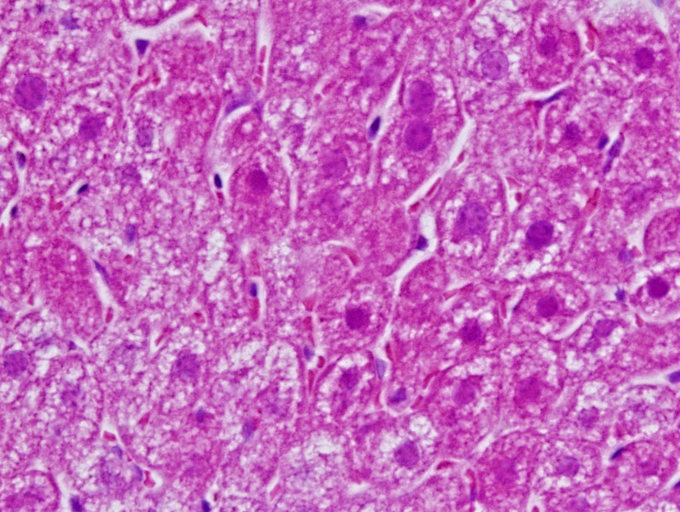

Supplement: Supplementary file 7 — Source data Fig. 6 [file 44319_2025_483_MOESM7_ESM.zip › Fig. 6 source data/Fig. 6C /Fig6C_p62S351A_low.tif]

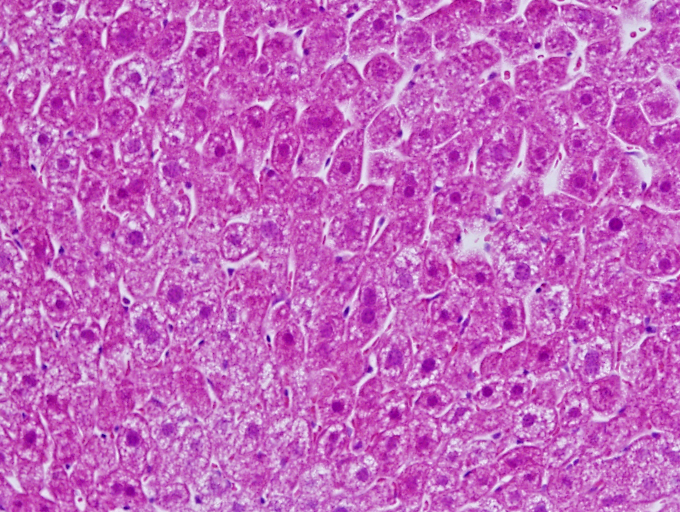

Supplement: Supplementary file 7 — Source data Fig. 6 [file 44319_2025_483_MOESM7_ESM.zip › Fig. 6 source data/Fig. 6C /Fig6C_p62S351A_up.tif]

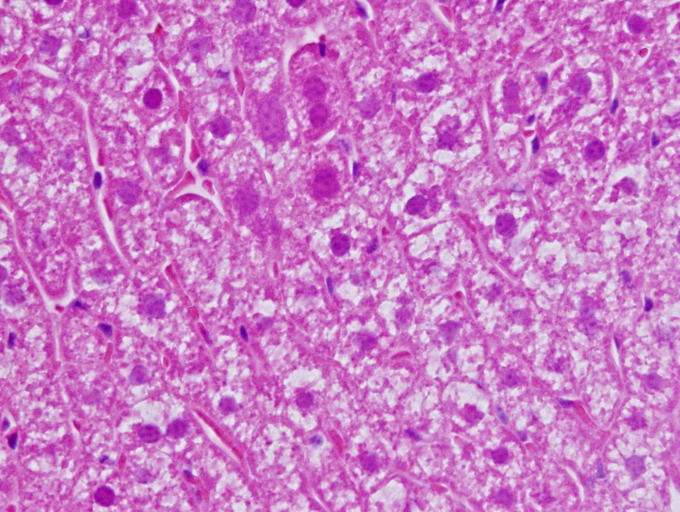

Supplement: Supplementary file 7 — Source data Fig. 6 [file 44319_2025_483_MOESM7_ESM.zip › Fig. 6 source data/Fig. 6C /Fig6C_cont_low.tif]

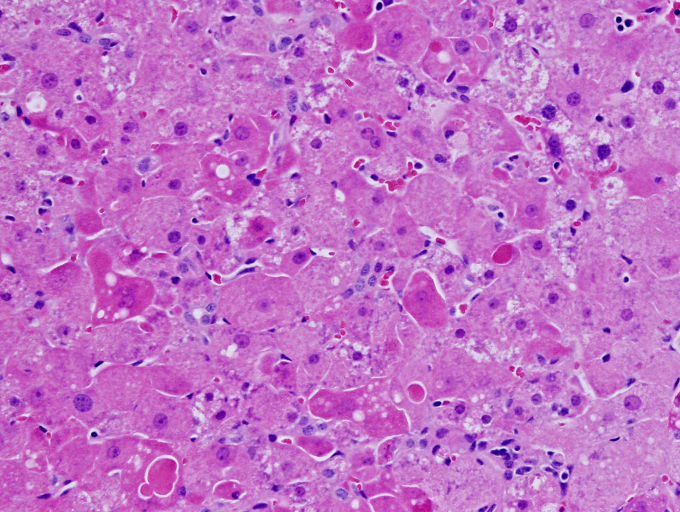

Supplement: Supplementary file 7 — Source data Fig. 6 [file 44319_2025_483_MOESM7_ESM.zip › Fig. 6 source data/Fig. 6C /Fig6C_Atg7KO_up.tif]

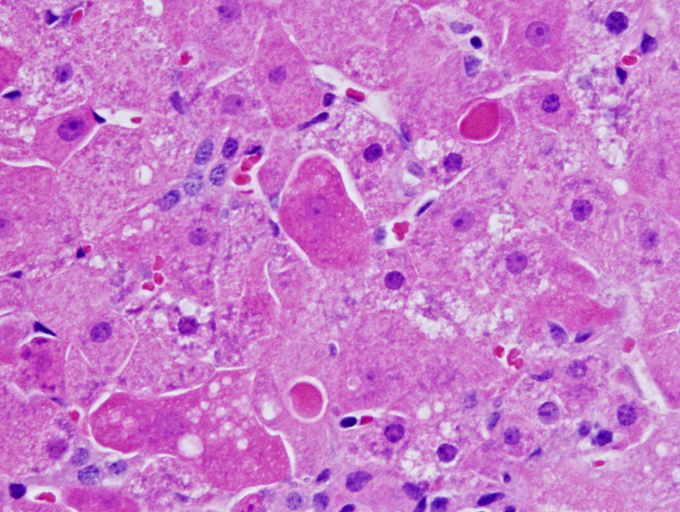

Supplement: Supplementary file 7 — Source data Fig. 6 [file 44319_2025_483_MOESM7_ESM.zip › Fig. 6 source data/Fig. 6C /Fig6C_Atg7KO_low.tif]

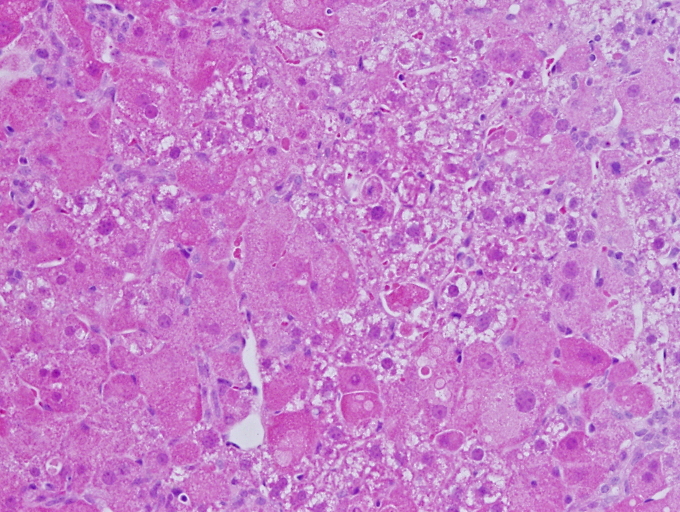

Supplement: Supplementary file 7 — Source data Fig. 6 [file 44319_2025_483_MOESM7_ESM.zip › Fig. 6 source data/Fig. 6C /Fig6C_Atg7KO p62S351A_up.tif]

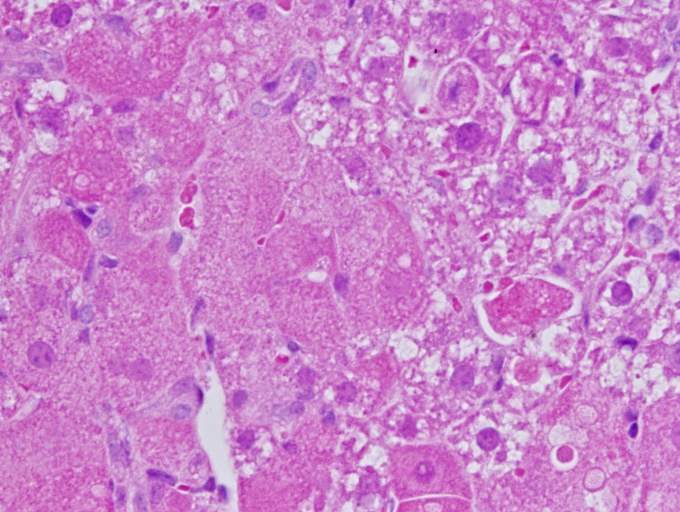

Supplement: Supplementary file 7 — Source data Fig. 6 [file 44319_2025_483_MOESM7_ESM.zip › Fig. 6 source data/Fig. 6C /Fig6C_Atg7KO p62S351A_low.tif]

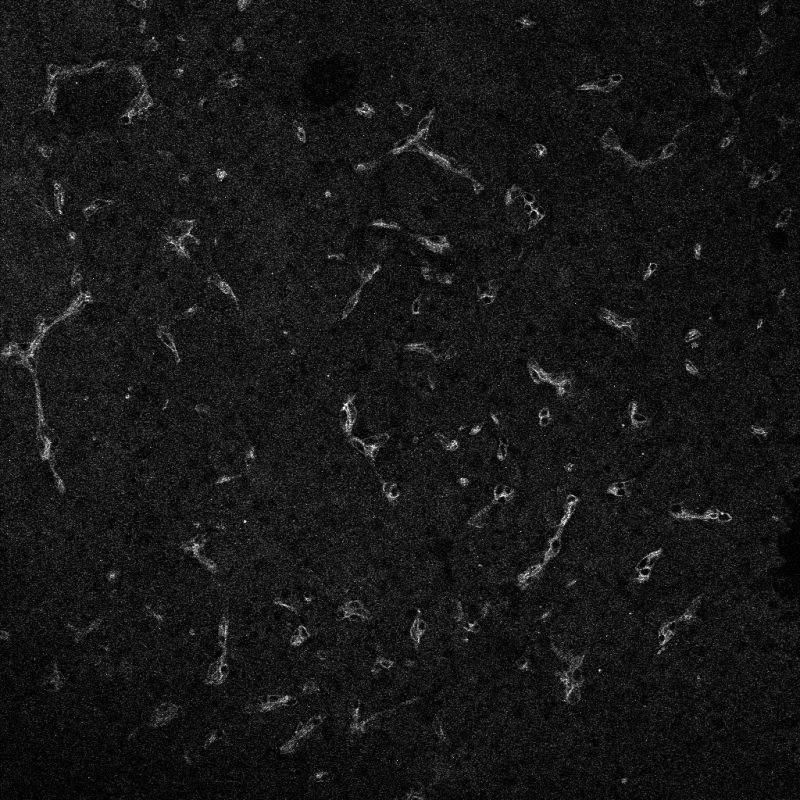

Supplement: Supplementary file 7 — Source data Fig. 6 [file 44319_2025_483_MOESM7_ESM.zip › Fig. 6 source data/Fig. 6F/Fig6F_Atg7KO_CK19.tif]

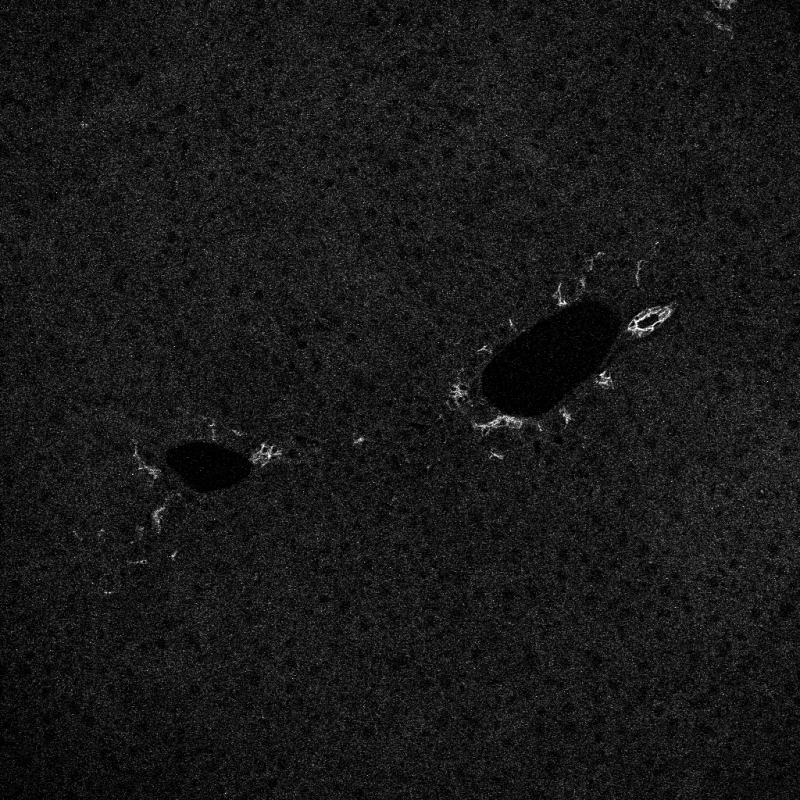

Supplement: Supplementary file 7 — Source data Fig. 6 [file 44319_2025_483_MOESM7_ESM.zip › Fig. 6 source data/Fig. 6F/Fig6F_p62T352A_CK19.tif]

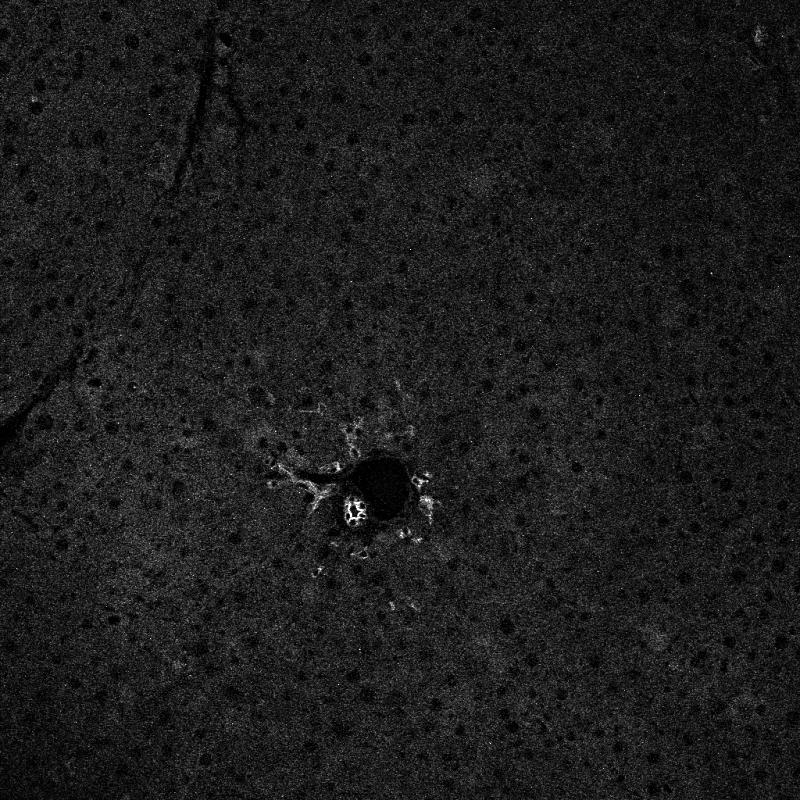

Supplement: Supplementary file 7 — Source data Fig. 6 [file 44319_2025_483_MOESM7_ESM.zip › Fig. 6 source data/Fig. 6F/Fig6F_Atg7KO p62T352A_CK19.tif]

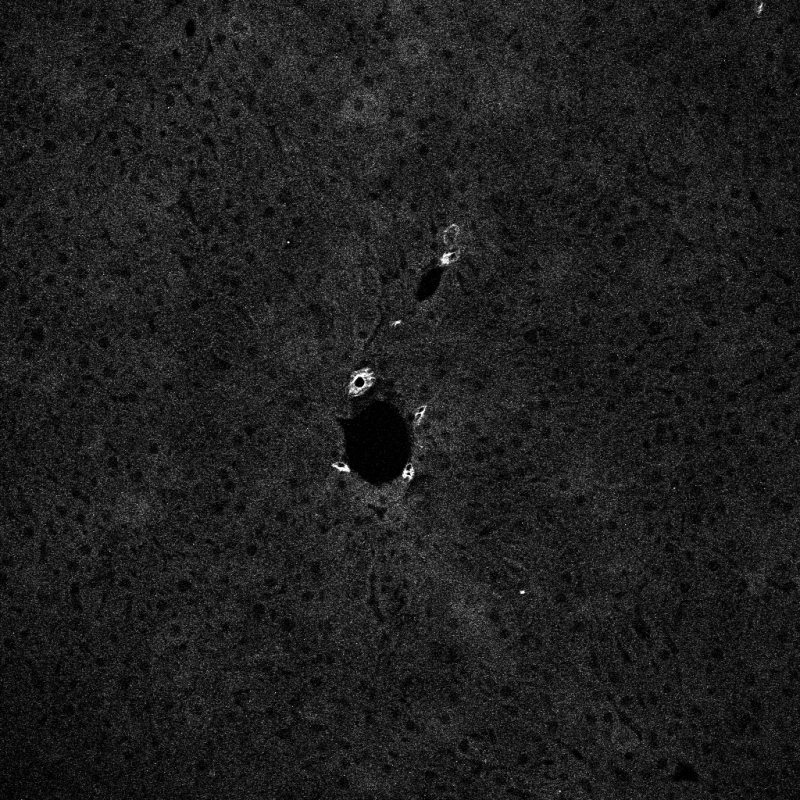

Supplement: Supplementary file 7 — Source data Fig. 6 [file 44319_2025_483_MOESM7_ESM.zip › Fig. 6 source data/Fig. 6F/Fig6F_cont_CK19.tif]

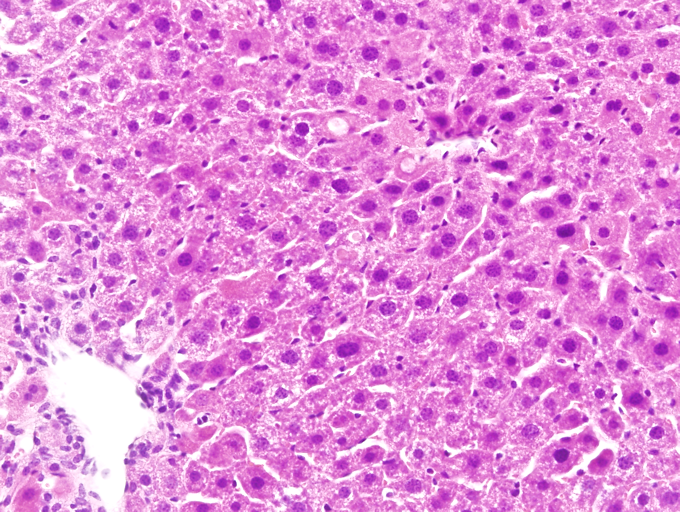

Supplement: Supplementary file 7 — Source data Fig. 6 [file 44319_2025_483_MOESM7_ESM.zip › Fig. 6 source data/Fig. 6D/Fig6D_Atg7KO_p62T352A_up.tif]

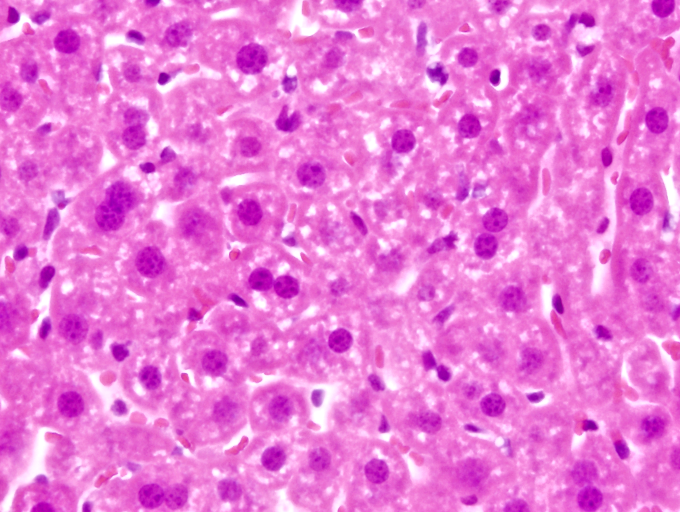

Supplement: Supplementary file 7 — Source data Fig. 6 [file 44319_2025_483_MOESM7_ESM.zip › Fig. 6 source data/Fig. 6D/Fig6D_T352A_low.tif]

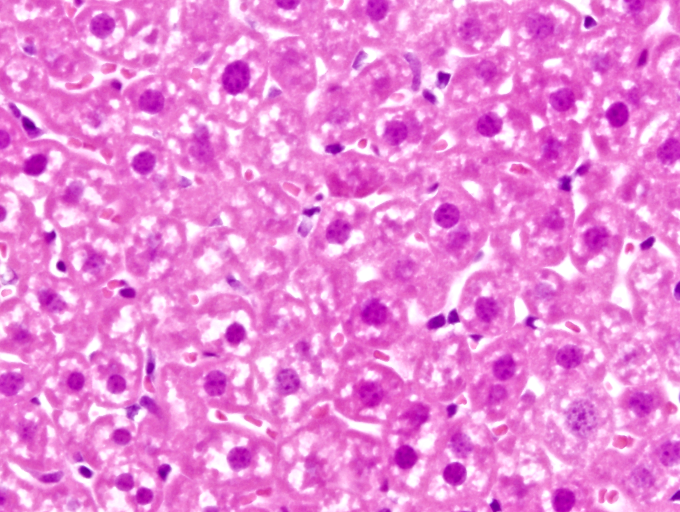

Supplement: Supplementary file 7 — Source data Fig. 6 [file 44319_2025_483_MOESM7_ESM.zip › Fig. 6 source data/Fig. 6D/Fig6D_cont_low.tif]

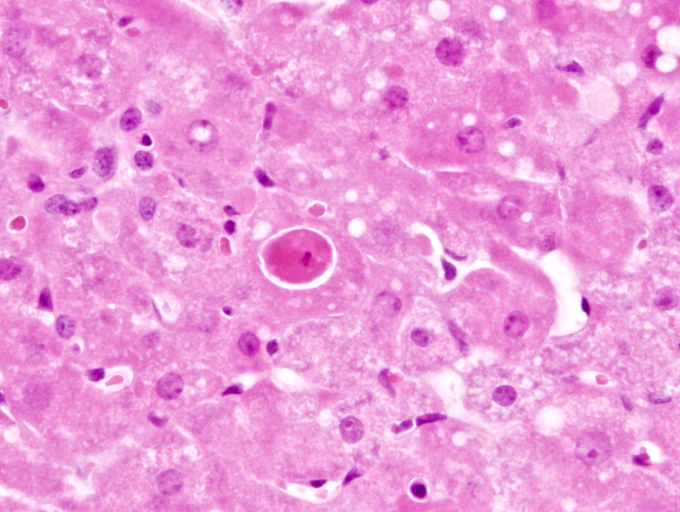

Supplement: Supplementary file 7 — Source data Fig. 6 [file 44319_2025_483_MOESM7_ESM.zip › Fig. 6 source data/Fig. 6D/Fig6D_Atg7KO_low.tif]

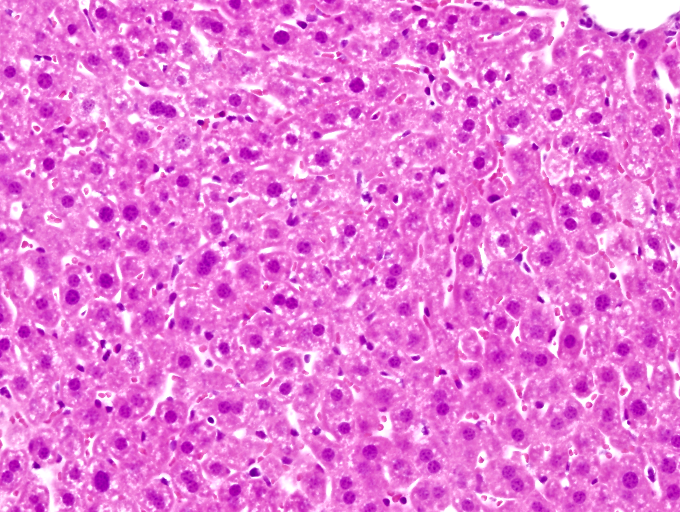

Supplement: Supplementary file 7 — Source data Fig. 6 [file 44319_2025_483_MOESM7_ESM.zip › Fig. 6 source data/Fig. 6D/Fig6D_T352A_up.tif]

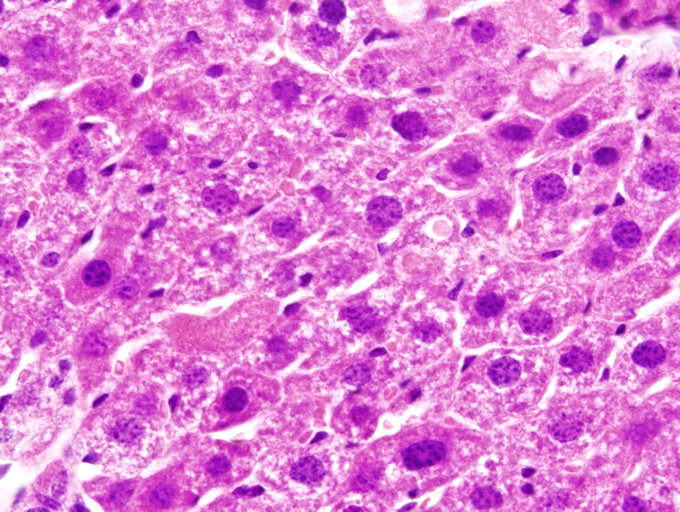

Supplement: Supplementary file 7 — Source data Fig. 6 [file 44319_2025_483_MOESM7_ESM.zip › Fig. 6 source data/Fig. 6D/Fig6D_Atg7KO_p62T352A_low.tif]

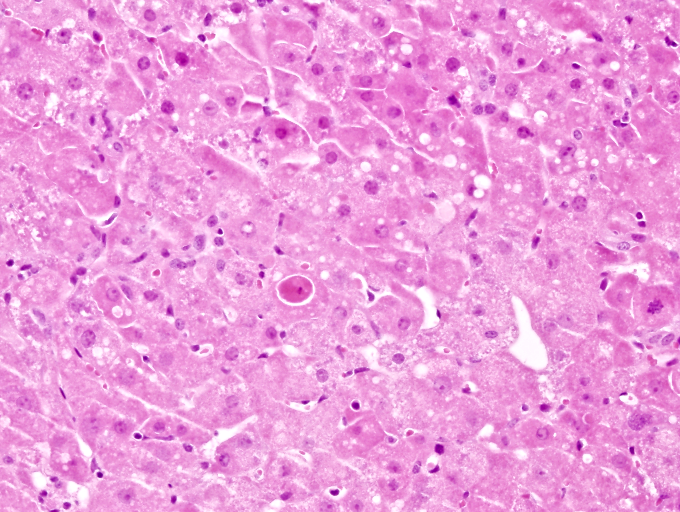

Supplement: Supplementary file 7 — Source data Fig. 6 [file 44319_2025_483_MOESM7_ESM.zip › Fig. 6 source data/Fig. 6D/Fig6D_Atg7KO_up.tif]

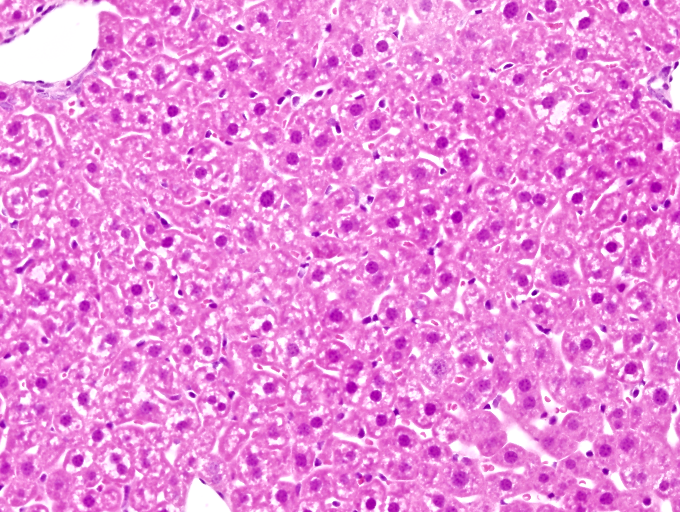

Supplement: Supplementary file 7 — Source data Fig. 6 [file 44319_2025_483_MOESM7_ESM.zip › Fig. 6 source data/Fig. 6D/Fig6D_cont_up.tif]

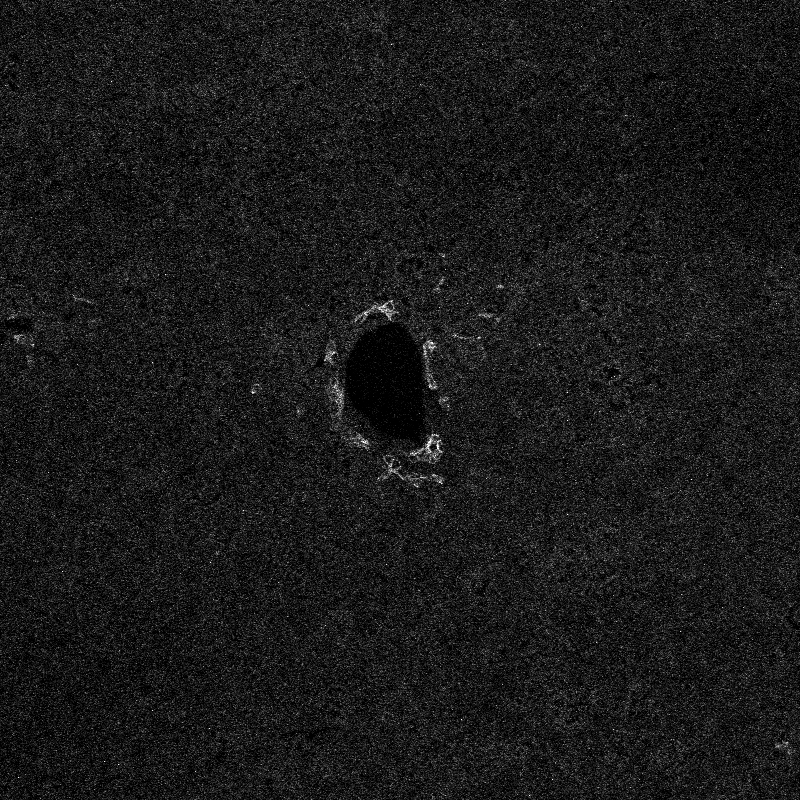

Supplement: Supplementary file 7 — Source data Fig. 6 [file 44319_2025_483_MOESM7_ESM.zip › Fig. 6 source data/Fig. 6E/Fig6E_cont_CK19.tif]

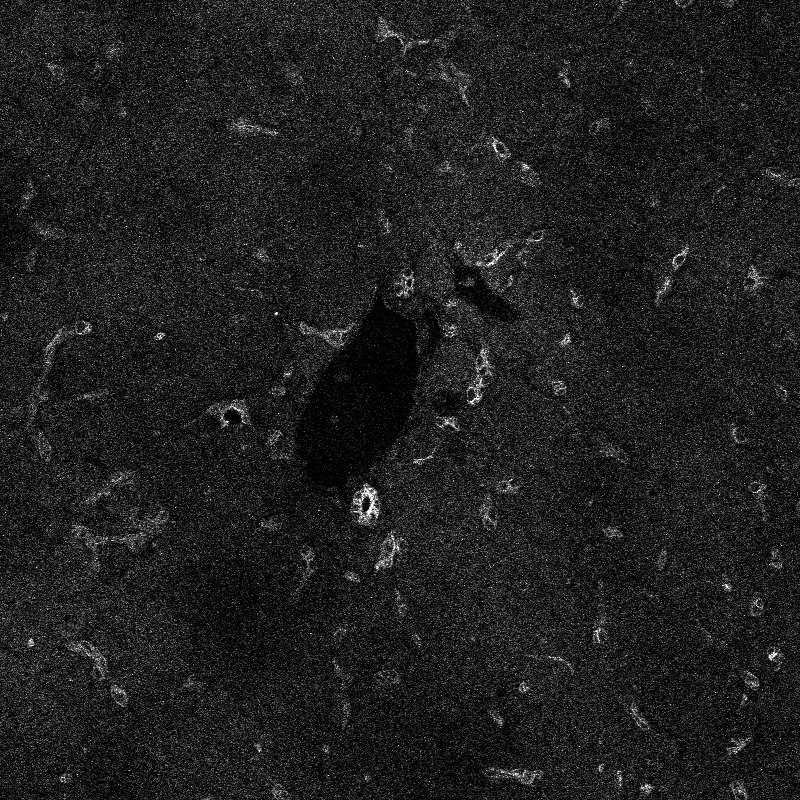

Supplement: Supplementary file 7 — Source data Fig. 6 [file 44319_2025_483_MOESM7_ESM.zip › Fig. 6 source data/Fig. 6E/Fig6E_Atg7KO_CK19.tif]

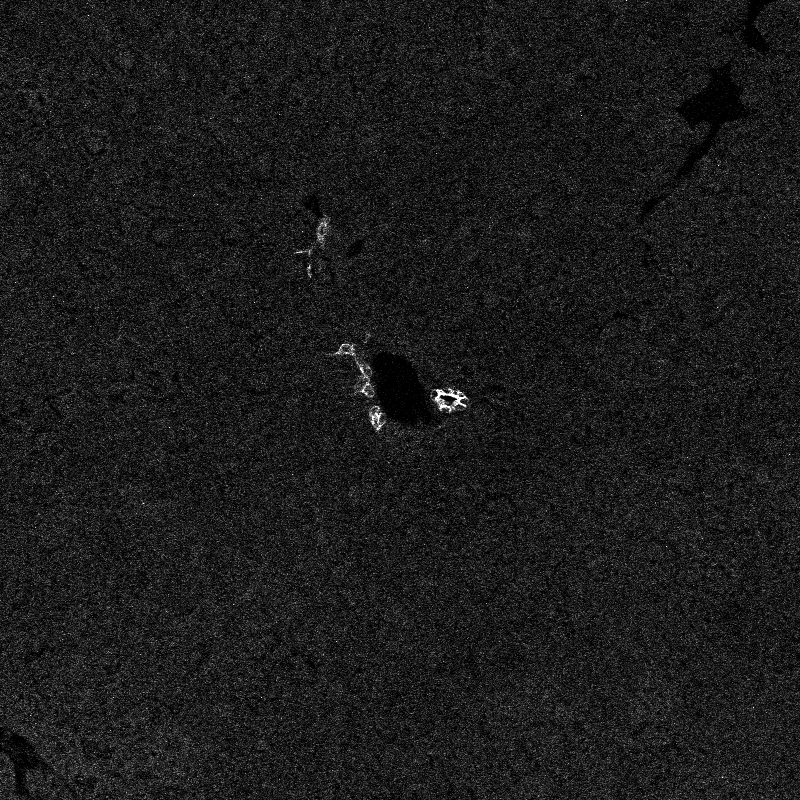

Supplement: Supplementary file 7 — Source data Fig. 6 [file 44319_2025_483_MOESM7_ESM.zip › Fig. 6 source data/Fig. 6E/Fig6E_p62S351A_CK19.tif]

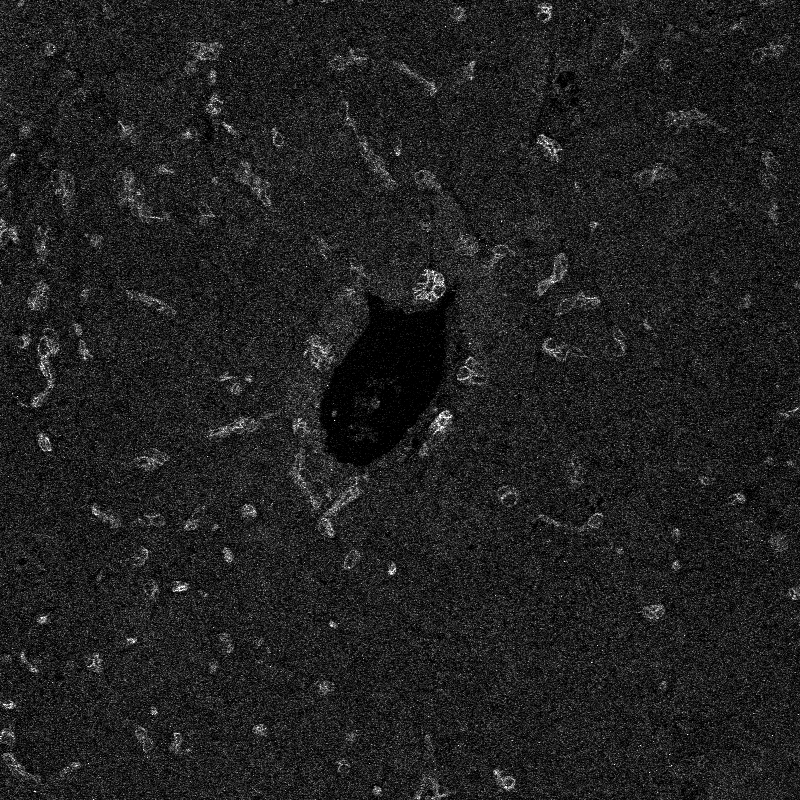

Supplement: Supplementary file 7 — Source data Fig. 6 [file 44319_2025_483_MOESM7_ESM.zip › Fig. 6 source data/Fig. 6E/Fig6E_Atg7KO p62S351A_CK19.tif]

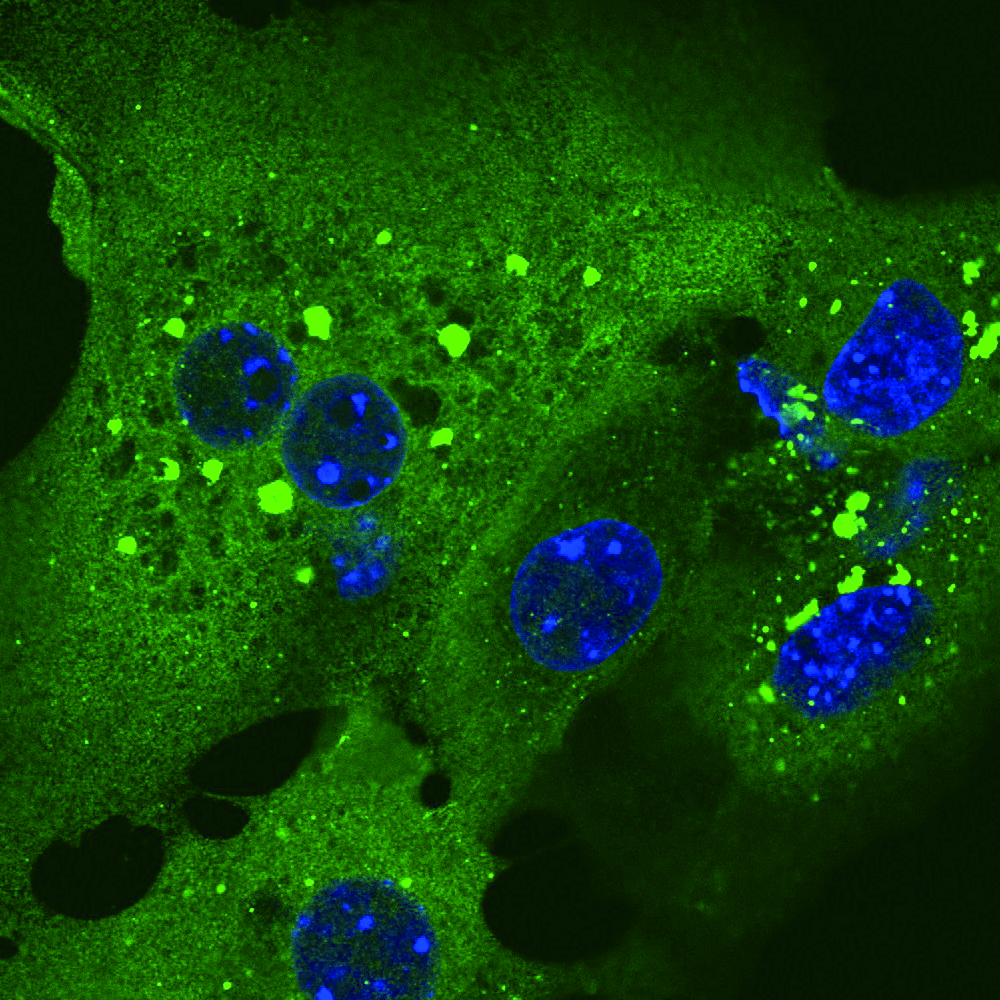

Supplement: Supplementary file 9 — Figure EV1 Source Data [file 44319_2025_483_MOESM9_ESM.zip › EV1 source data/EV1D/EV1D_Atg7C572S_24h.tif]

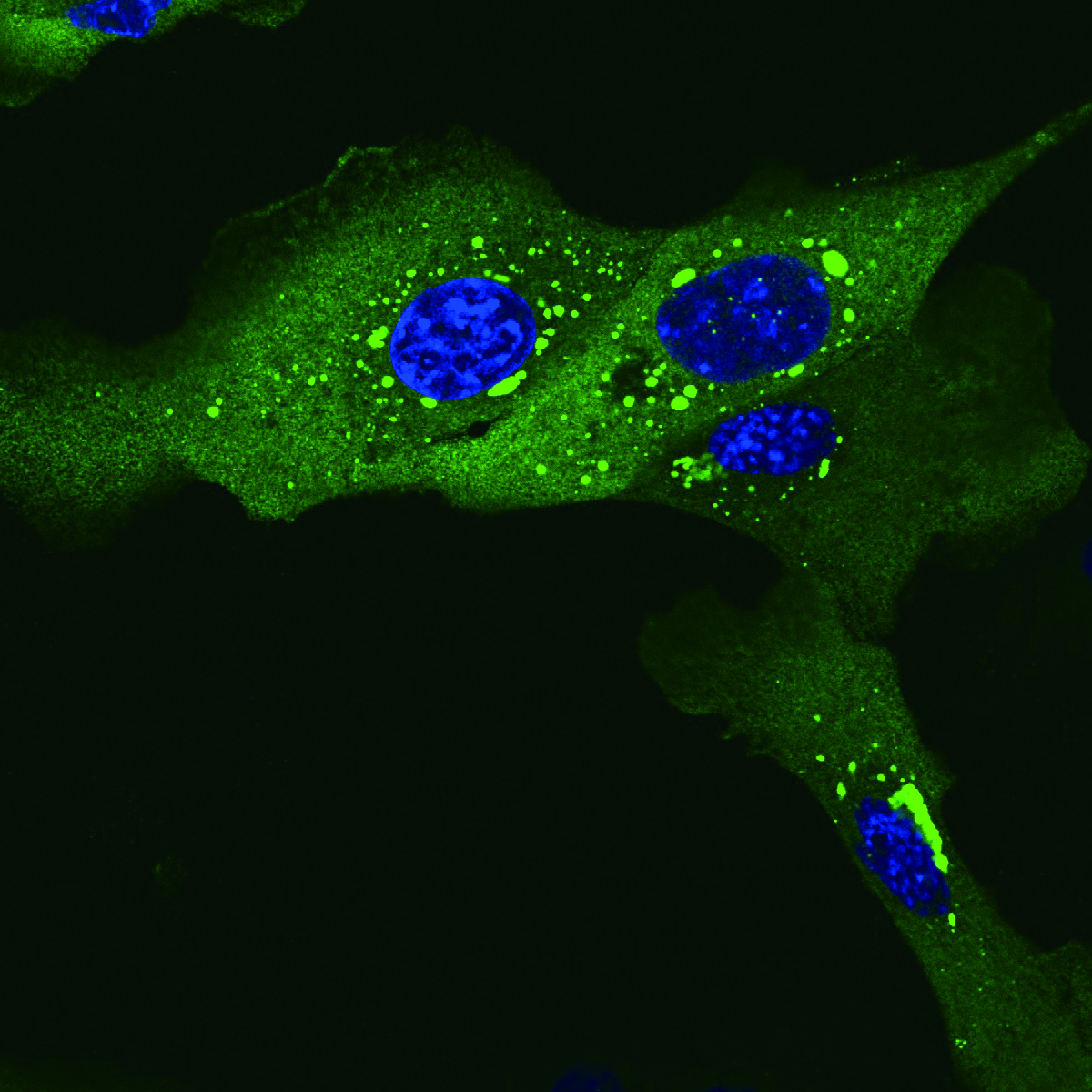

Supplement: Supplementary file 9 — Figure EV1 Source Data [file 44319_2025_483_MOESM9_ESM.zip › EV1 source data/EV1D/EV1D_Atg7C572S_12h.tif]

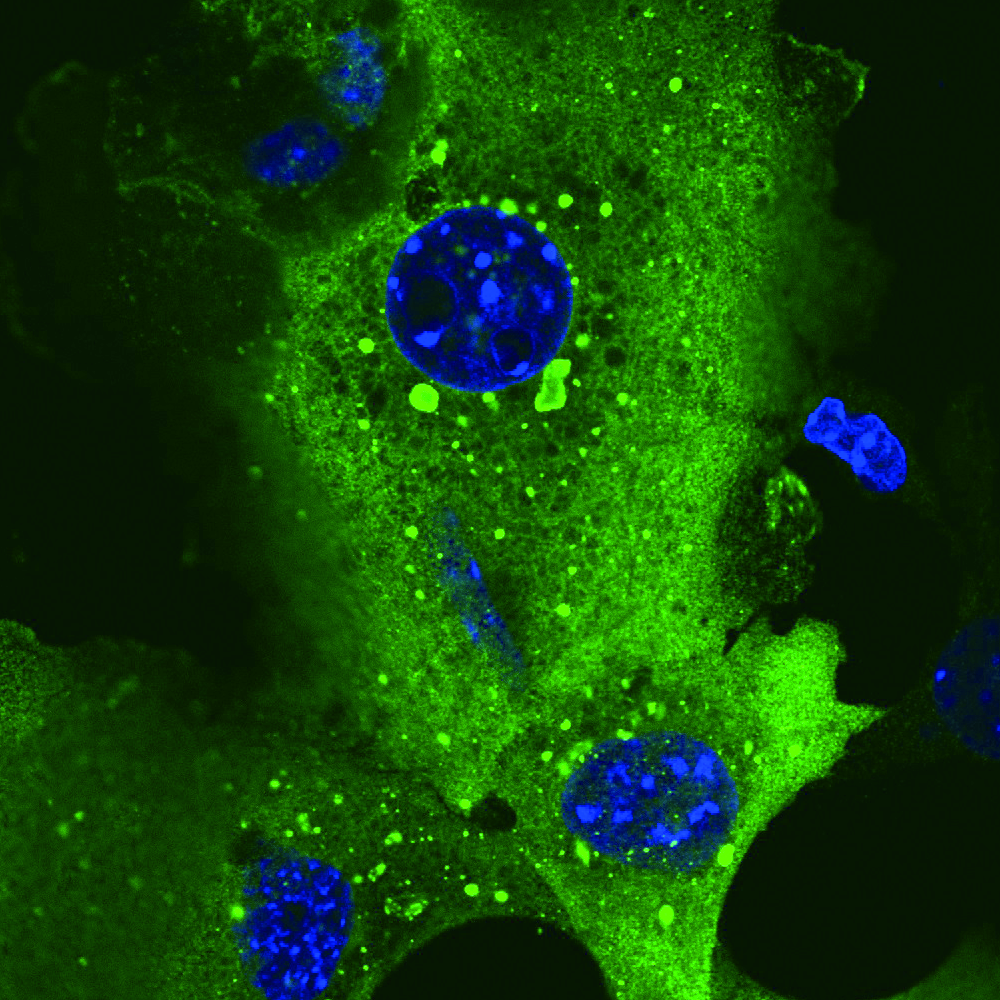

Supplement: Supplementary file 9 — Figure EV1 Source Data [file 44319_2025_483_MOESM9_ESM.zip › EV1 source data/EV1D/EV1D_Atg7C572S_48h.tif]

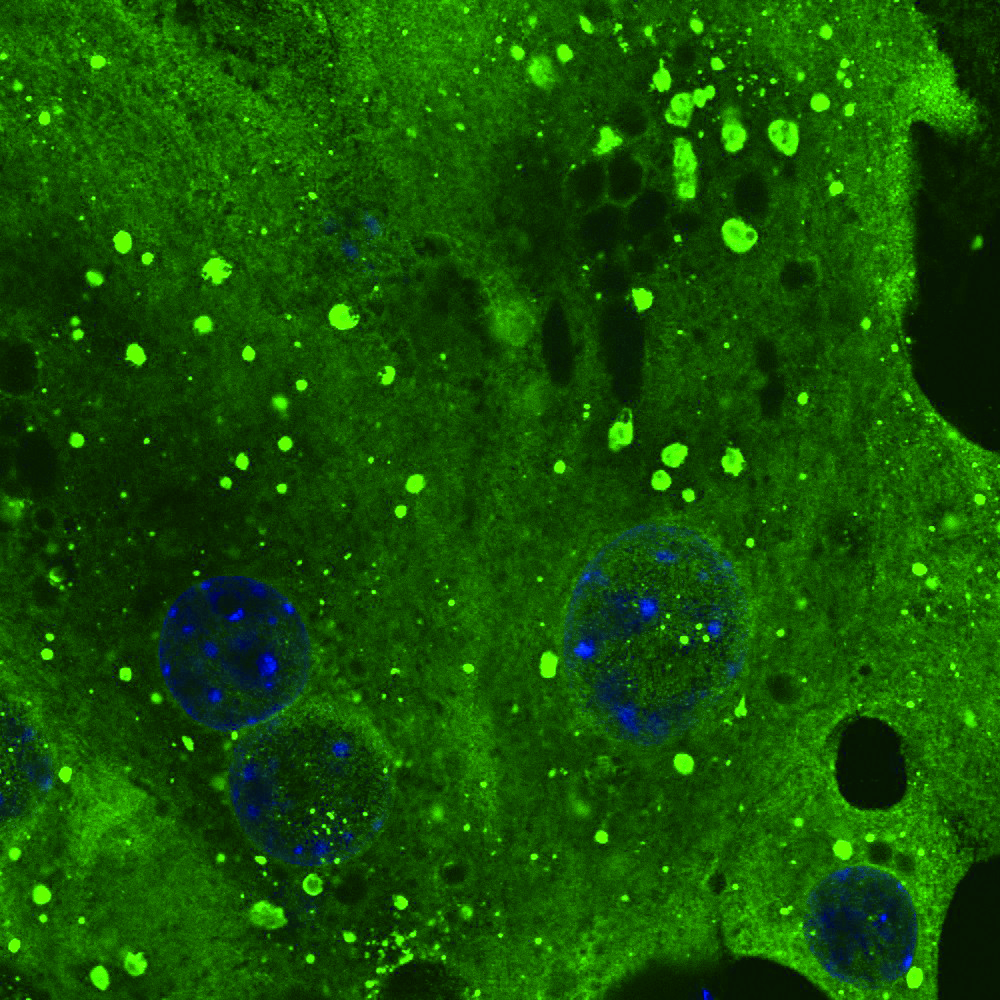

Supplement: Supplementary file 9 — Figure EV1 Source Data [file 44319_2025_483_MOESM9_ESM.zip › EV1 source data/EV1D/EV1D_Atg7WT_12h.tif]

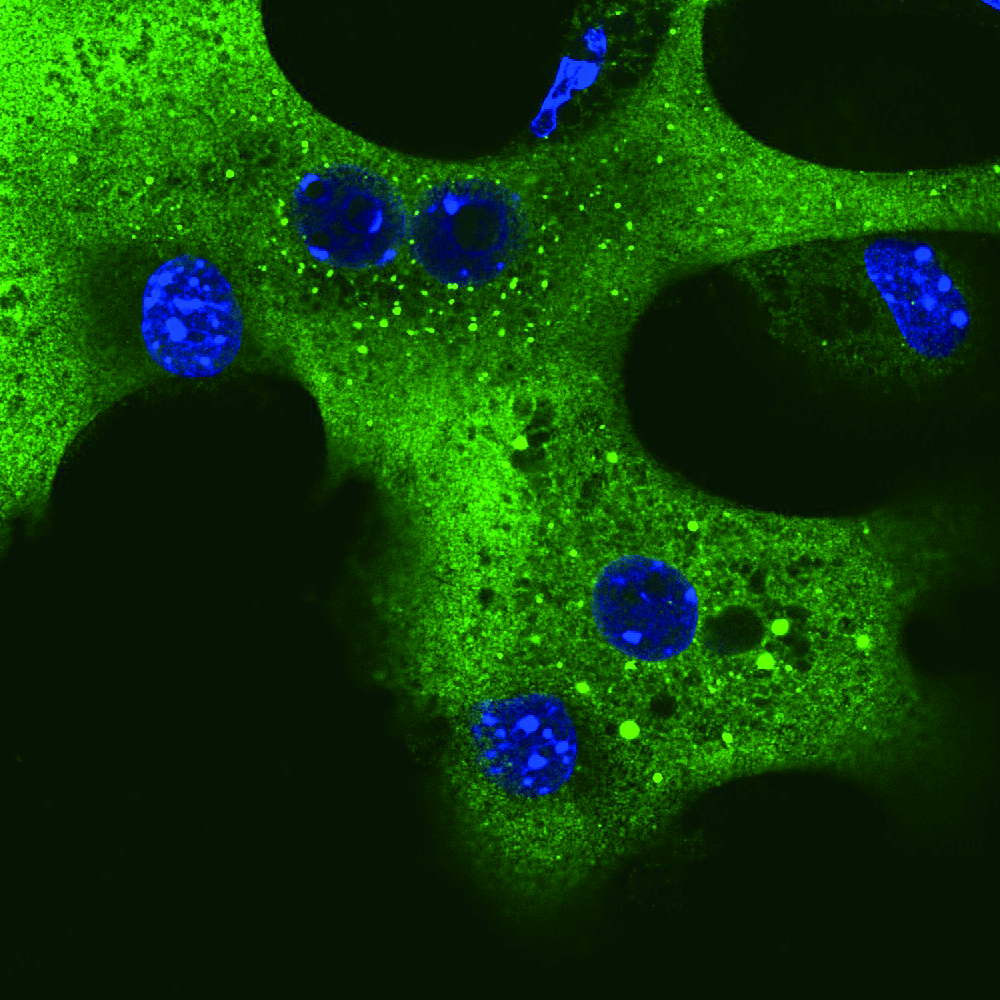

Supplement: Supplementary file 9 — Figure EV1 Source Data [file 44319_2025_483_MOESM9_ESM.zip › EV1 source data/EV1D/EV1D_Atg7WT_0h.tif]

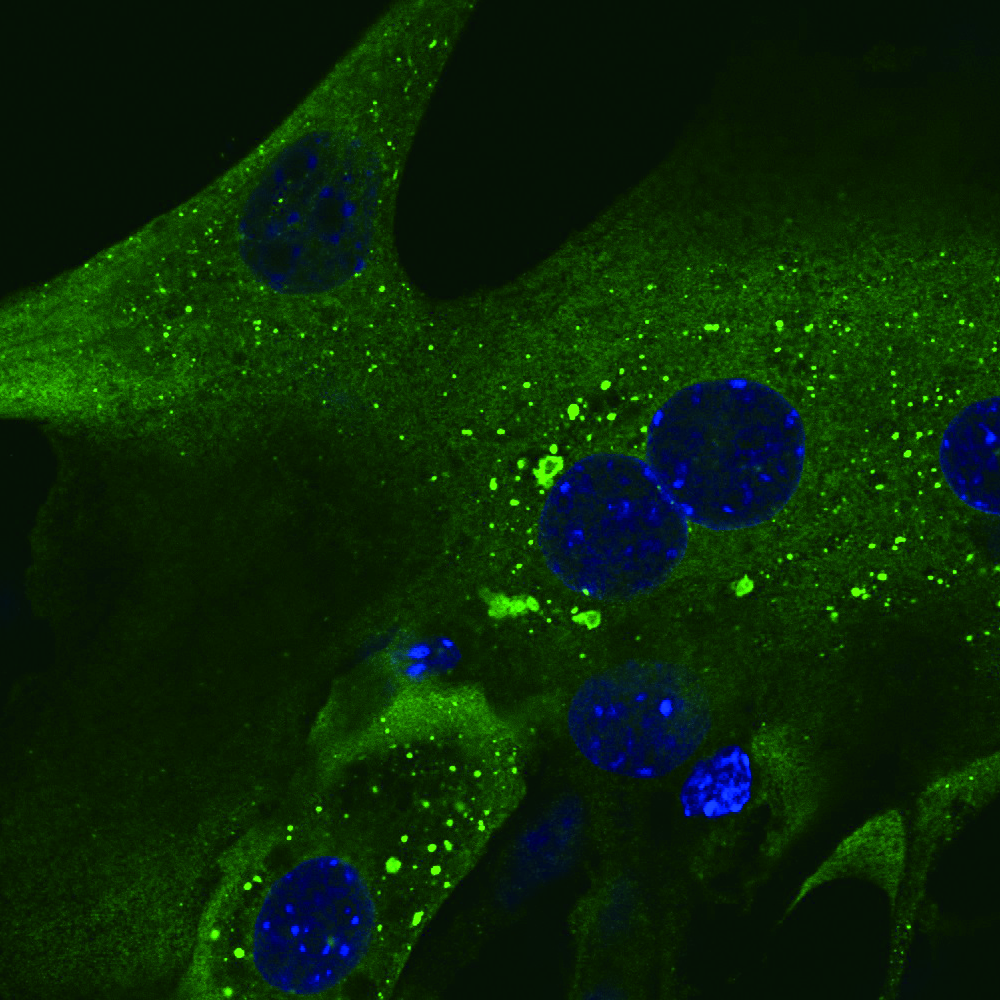

Supplement: Supplementary file 9 — Figure EV1 Source Data [file 44319_2025_483_MOESM9_ESM.zip › EV1 source data/EV1D/EV1D_Atg7WT_24h.tif]

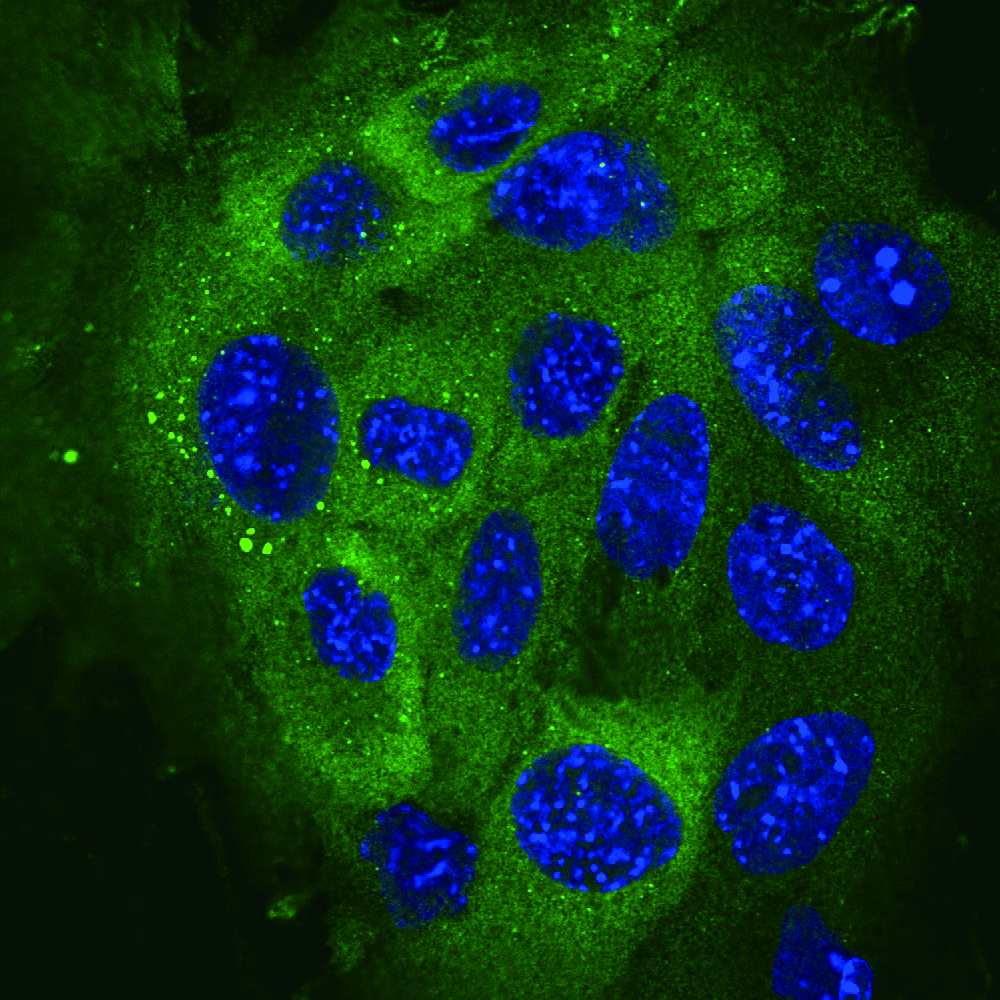

Supplement: Supplementary file 9 — Figure EV1 Source Data [file 44319_2025_483_MOESM9_ESM.zip › EV1 source data/EV1D/EV1D_Atg7WT_48h.tif]

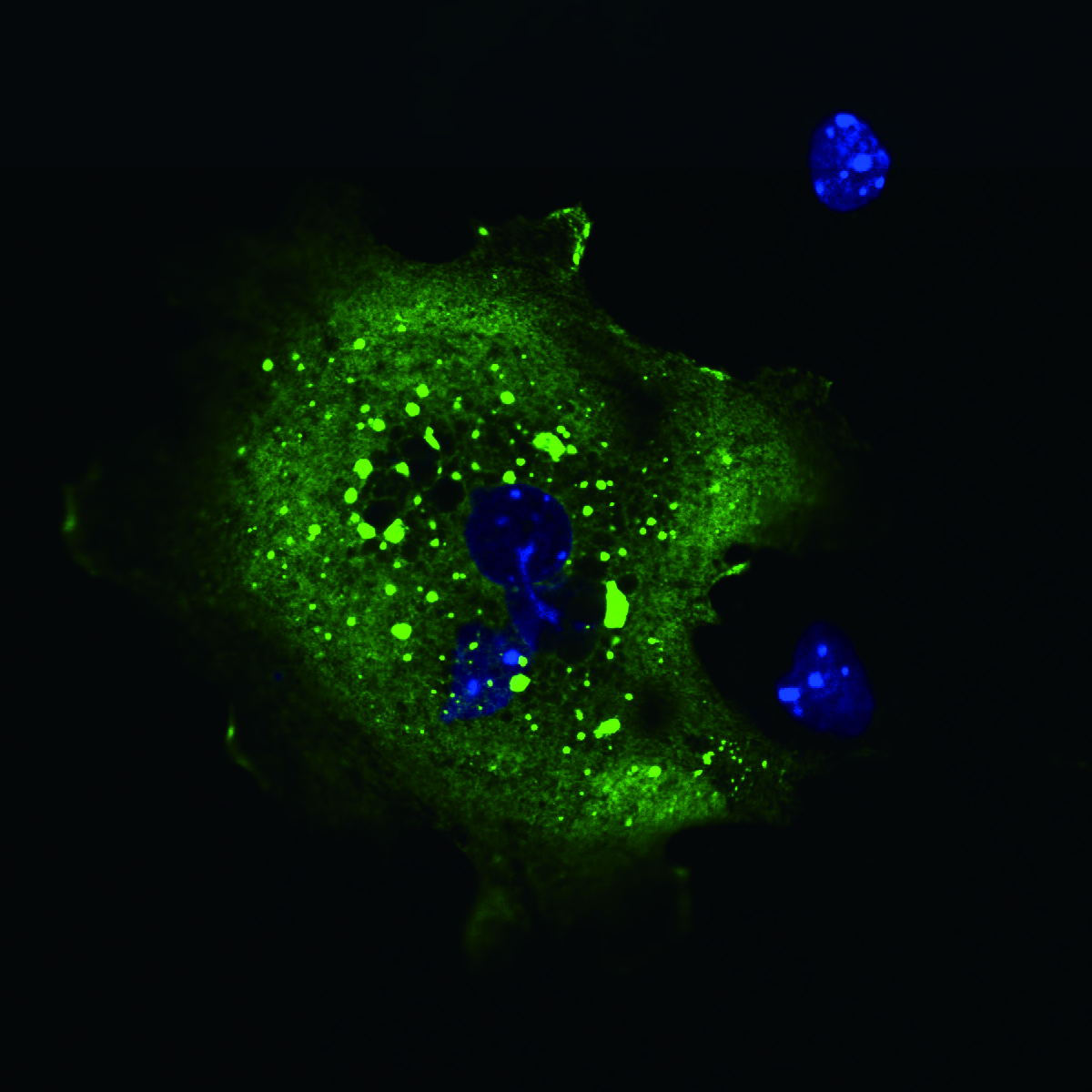

Supplement: Supplementary file 9 — Figure EV1 Source Data [file 44319_2025_483_MOESM9_ESM.zip › EV1 source data/EV1D/EV1D_Atg7C572S_0h.tif]

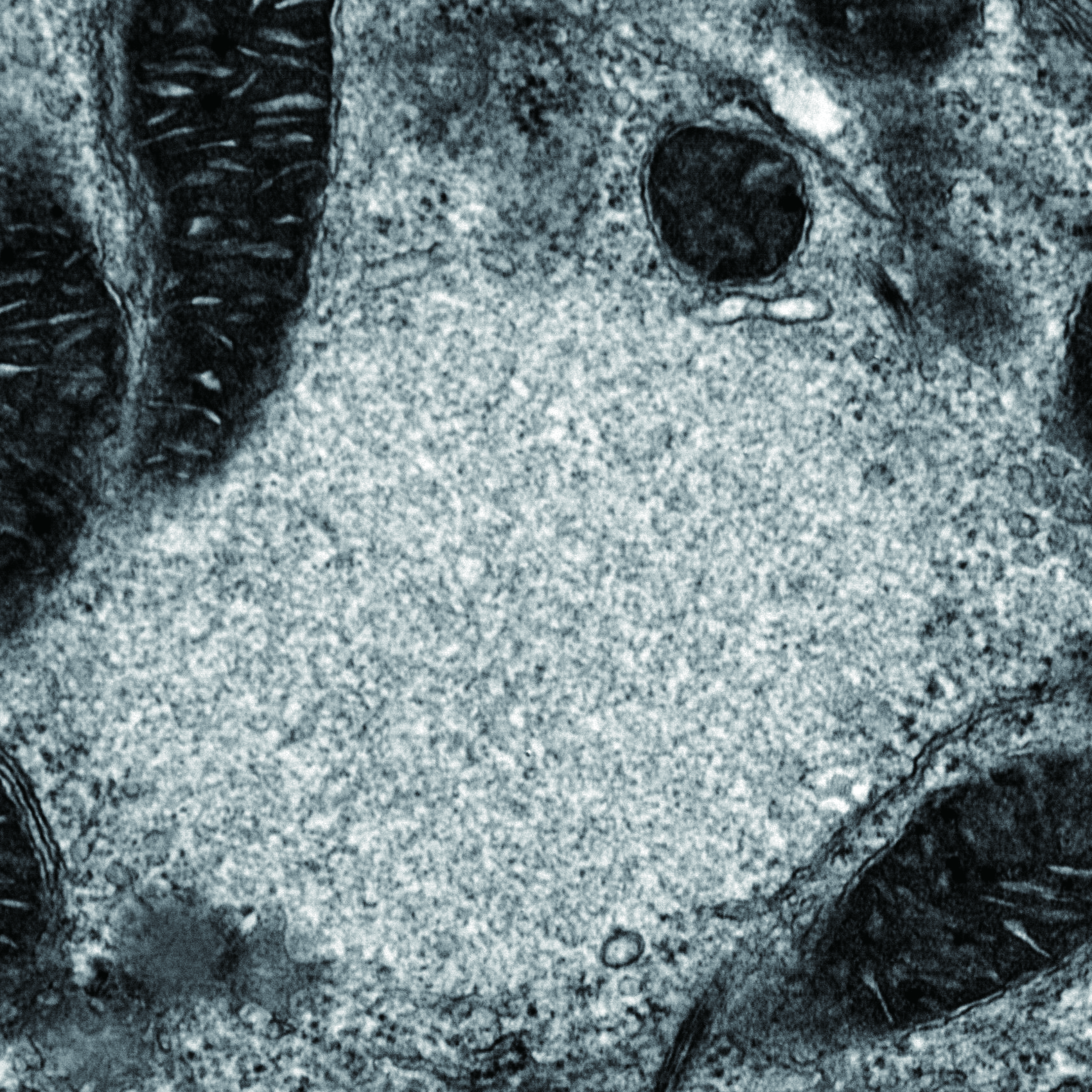

Supplement: Supplementary file 9 — Figure EV1 Source Data [file 44319_2025_483_MOESM9_ESM.zip › EV1 source data/EV1B/EV1B_Atg7KO_HM.tif]

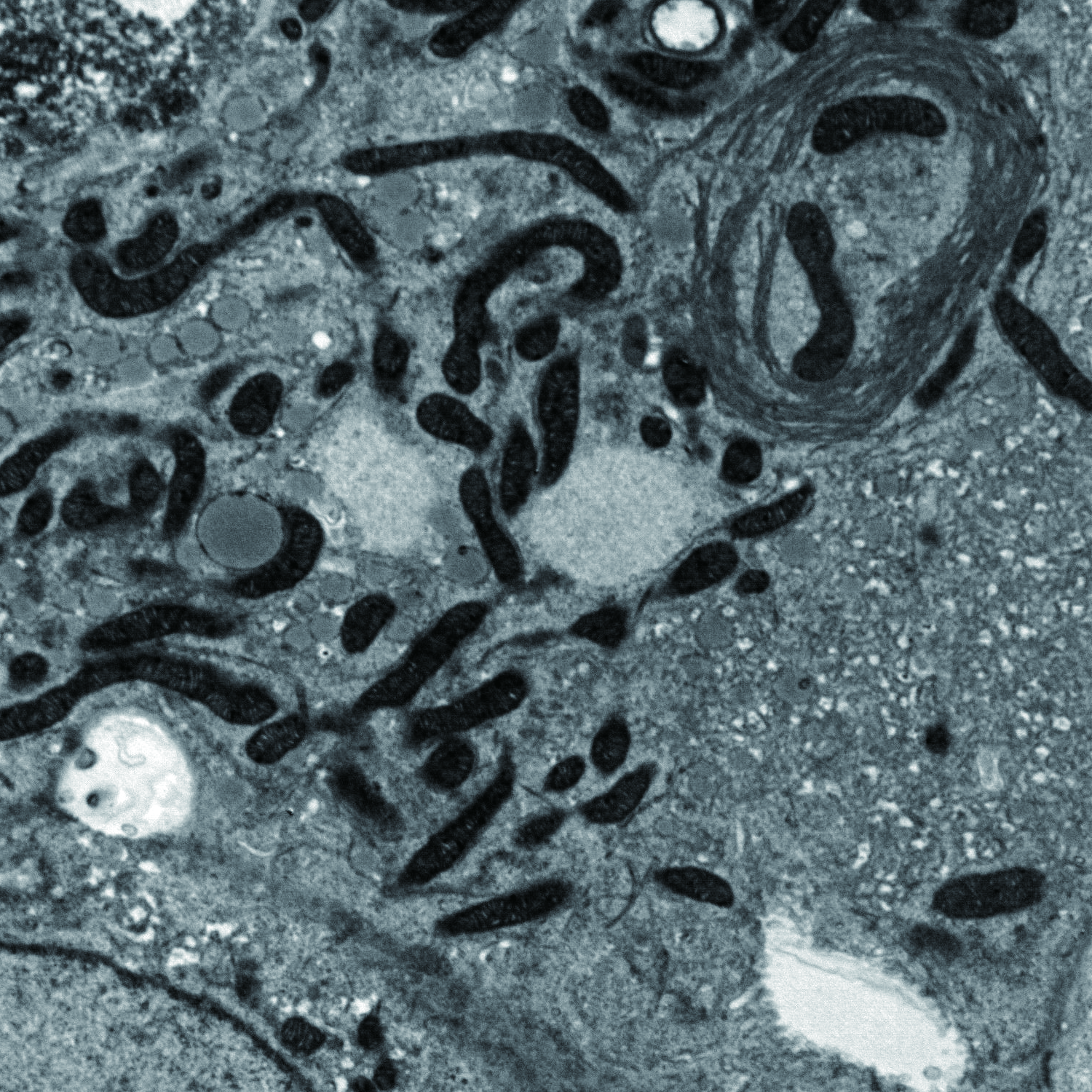

Supplement: Supplementary file 9 — Figure EV1 Source Data [file 44319_2025_483_MOESM9_ESM.zip › EV1 source data/EV1B/EV1B_Atg7KO.tif]

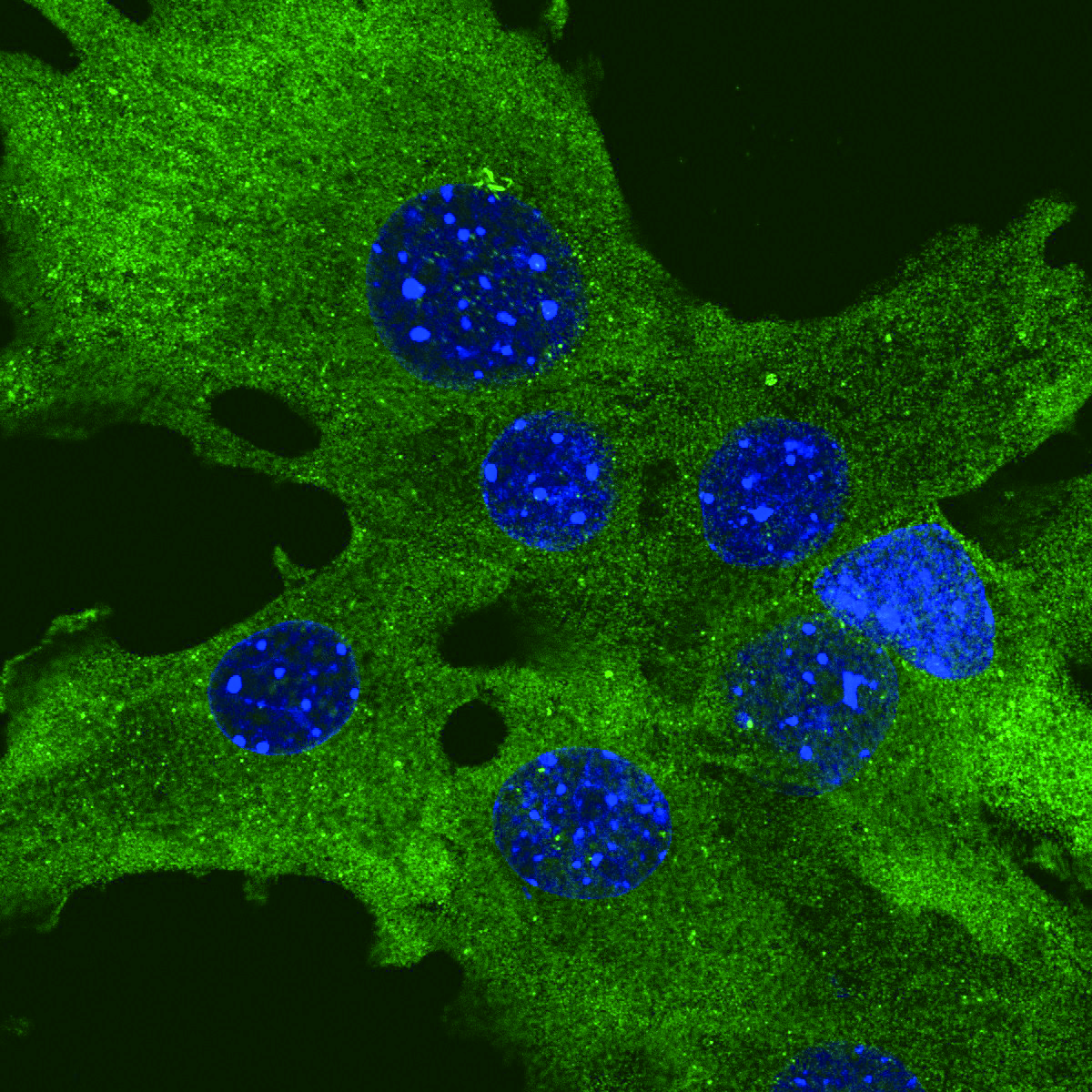

Supplement: Supplementary file 9 — Figure EV1 Source Data [file 44319_2025_483_MOESM9_ESM.zip › EV1 source data/EV1A/EV1A_Atg7FF.tif]

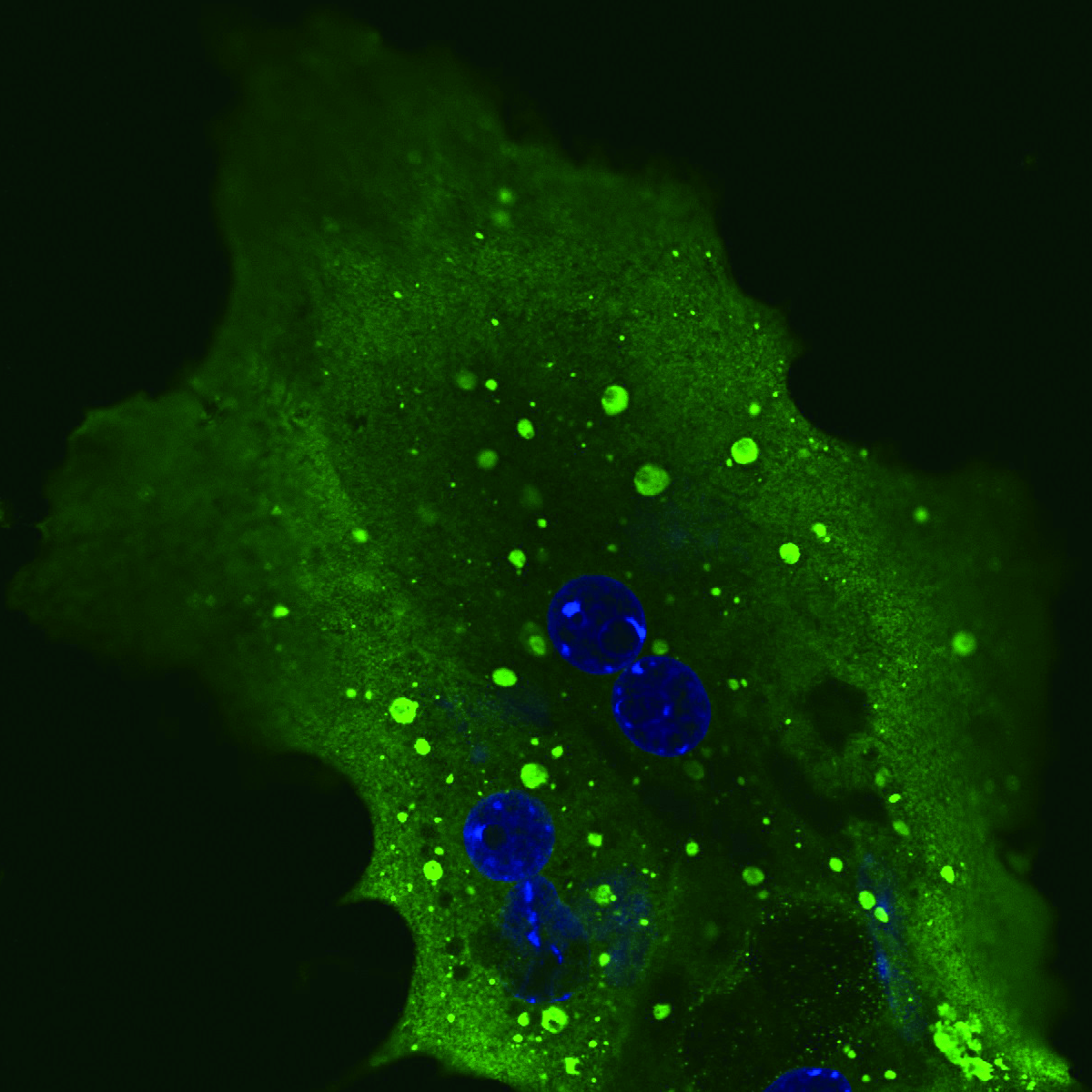

Supplement: Supplementary file 9 — Figure EV1 Source Data [file 44319_2025_483_MOESM9_ESM.zip › EV1 source data/EV1A/EV1A_Atg7KO.tif]

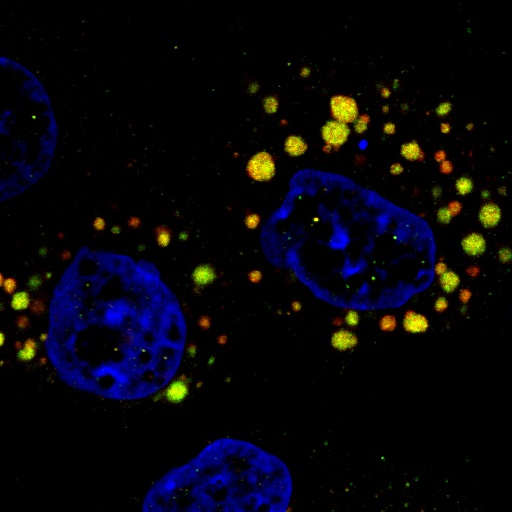

Supplement: Supplementary file 10 — Figure EV2 Source Data [file 44319_2025_483_MOESM10_ESM.zip › EV2 source data/EV2D/FIP200p62DKO-p62SE-KEAP1_Merged.jpg]

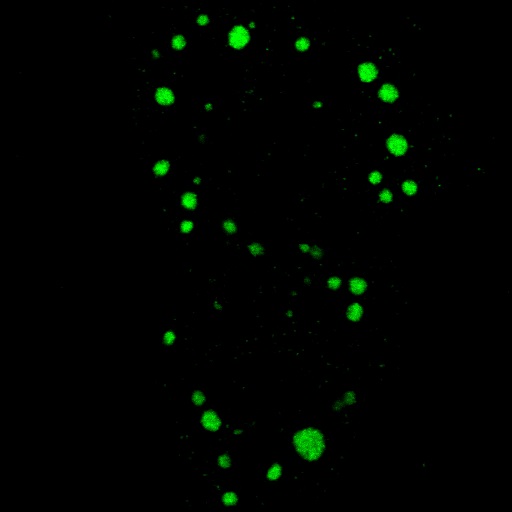

Supplement: Supplementary file 10 — Figure EV2 Source Data [file 44319_2025_483_MOESM10_ESM.zip › EV2 source data/EV2D/FIP200p62DKO-p62wt-KEAP1_KEAP1.jpg]

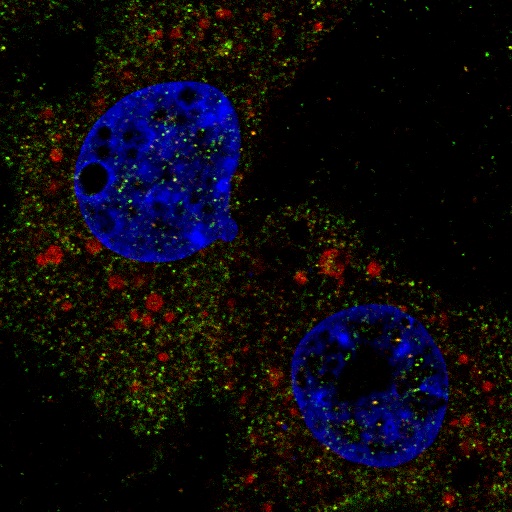

Supplement: Supplementary file 10 — Figure EV2 Source Data [file 44319_2025_483_MOESM10_ESM.zip › EV2 source data/EV2D/FIP200p62DKO-p62TA-KEAP1_Merged.jpg]

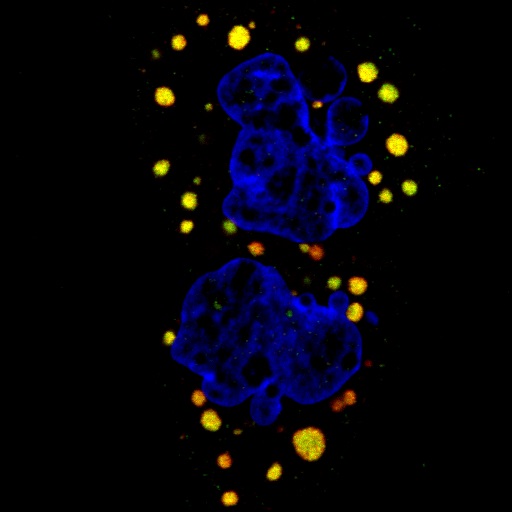

Supplement: Supplementary file 10 — Figure EV2 Source Data [file 44319_2025_483_MOESM10_ESM.zip › EV2 source data/EV2D/FIP200p62DKO-p62wt-KEAP1_Merged.jpg]

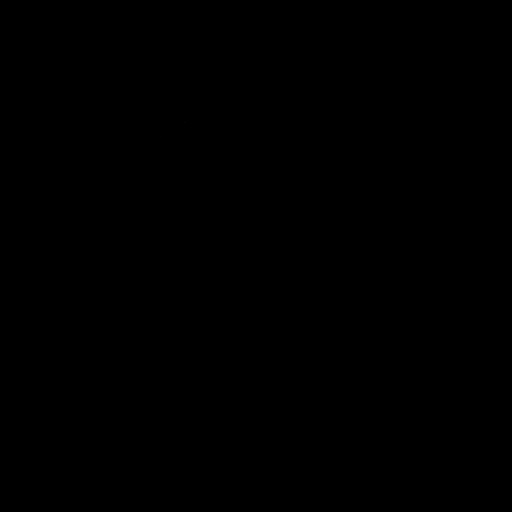

Supplement: Supplementary file 10 — Figure EV2 Source Data [file 44319_2025_483_MOESM10_ESM.zip › EV2 source data/EV2D/FIP200p62DKO-empty vector-KEAP1_p62.jpg]

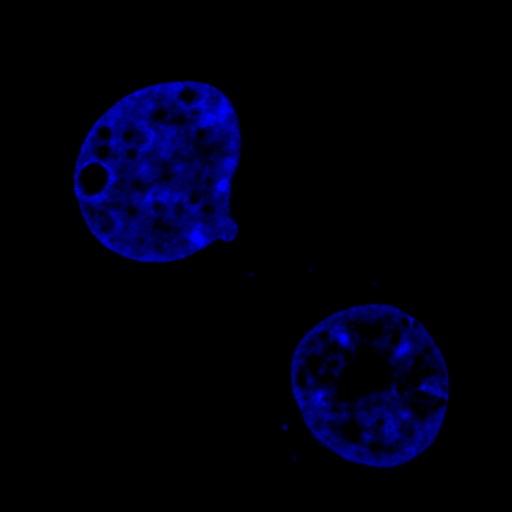

Supplement: Supplementary file 10 — Figure EV2 Source Data [file 44319_2025_483_MOESM10_ESM.zip › EV2 source data/EV2D/FIP200p62DKO-p62TA-KEAP1_DAPI.jpg]

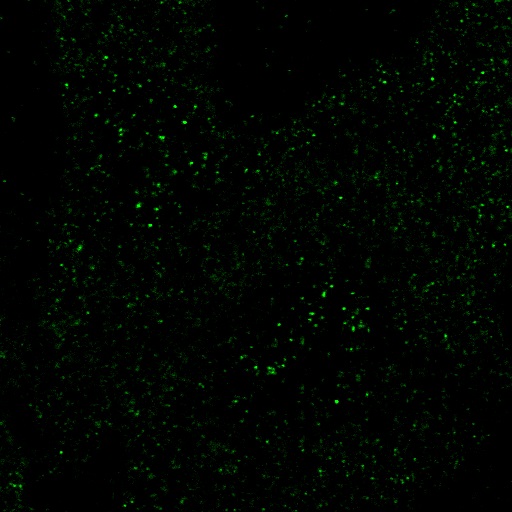

Supplement: Supplementary file 10 — Figure EV2 Source Data [file 44319_2025_483_MOESM10_ESM.zip › EV2 source data/EV2D/FIP200p62DKO-empty vector-KEAP1_KEAP1.jpg]

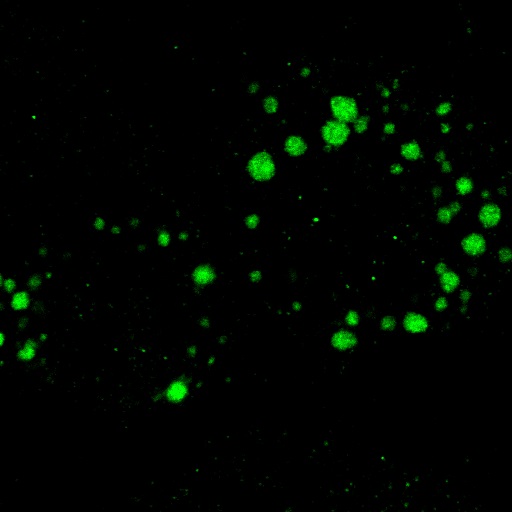

Supplement: Supplementary file 10 — Figure EV2 Source Data [file 44319_2025_483_MOESM10_ESM.zip › EV2 source data/EV2D/FIP200p62DKO-p62SE-KEAP1_KEAP1.jpg]

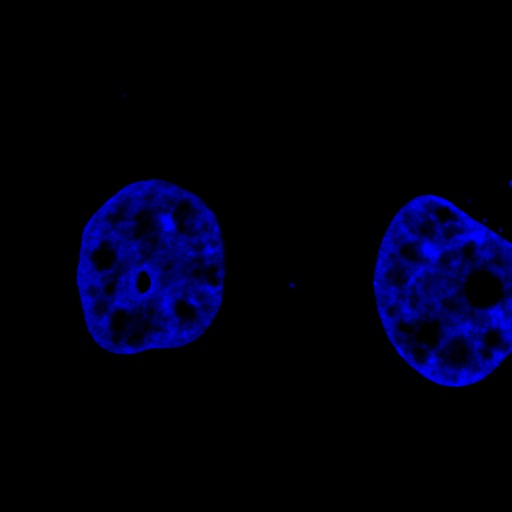

Supplement: Supplementary file 10 — Figure EV2 Source Data [file 44319_2025_483_MOESM10_ESM.zip › EV2 source data/EV2D/FIP200p62DKO-SA-KEAP1_DAPI.jpg]

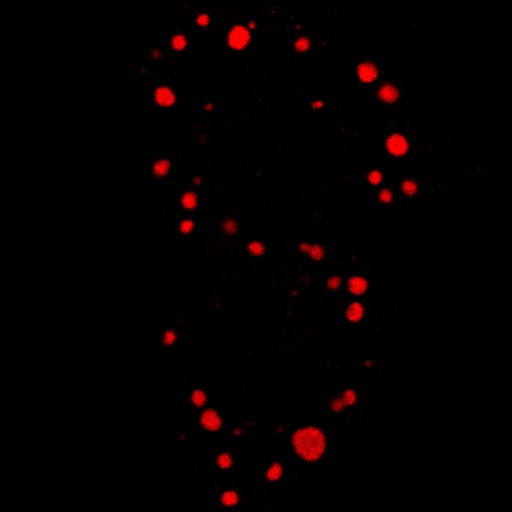

Supplement: Supplementary file 10 — Figure EV2 Source Data [file 44319_2025_483_MOESM10_ESM.zip › EV2 source data/EV2D/FIP200p62DKO-p62wt-KEAP1_p62.jpg]

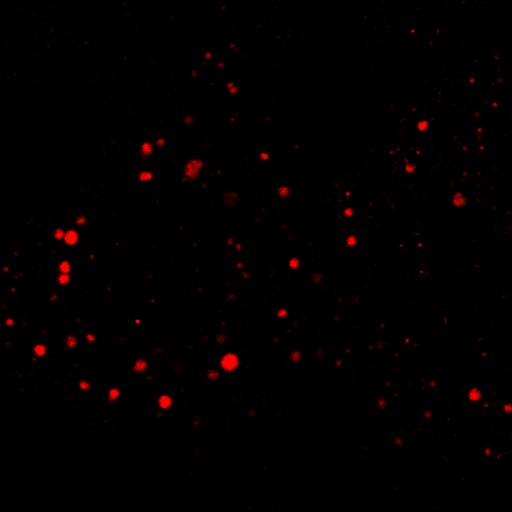

Supplement: Supplementary file 10 — Figure EV2 Source Data [file 44319_2025_483_MOESM10_ESM.zip › EV2 source data/EV2D/FIP200p62DKO-SA-KEAP1_p62.jpg]

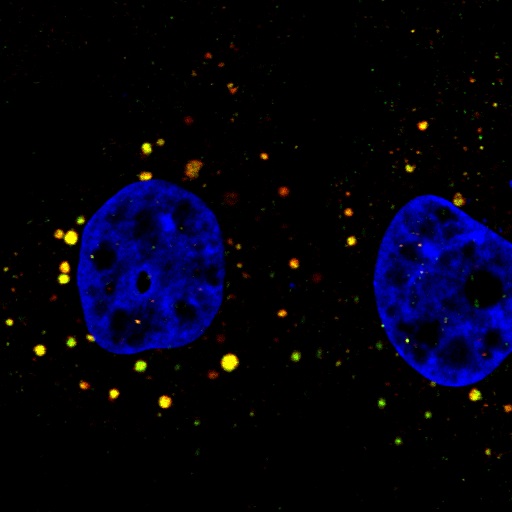

Supplement: Supplementary file 10 — Figure EV2 Source Data [file 44319_2025_483_MOESM10_ESM.zip › EV2 source data/EV2D/FIP200p62DKO-SA-KEAP1_Merged.jpg]

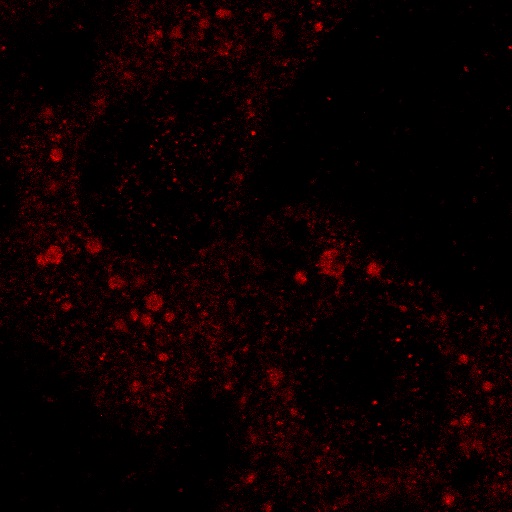

Supplement: Supplementary file 10 — Figure EV2 Source Data [file 44319_2025_483_MOESM10_ESM.zip › EV2 source data/EV2D/FIP200p62DKO-p62TA-KEAP1_p62.jpg]

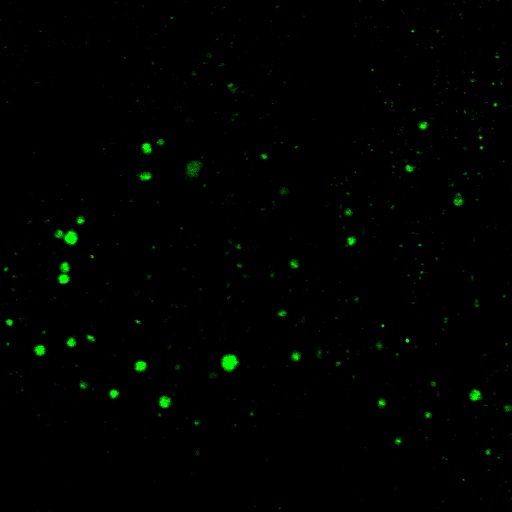

Supplement: Supplementary file 10 — Figure EV2 Source Data [file 44319_2025_483_MOESM10_ESM.zip › EV2 source data/EV2D/FIP200p62DKO-SA-KEAP1_KEAP1.jpg]

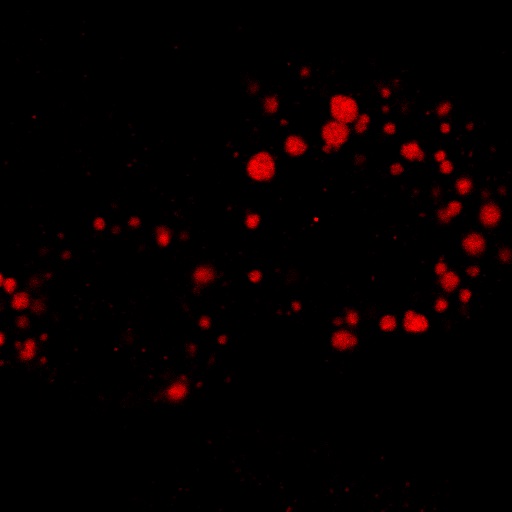

Supplement: Supplementary file 10 — Figure EV2 Source Data [file 44319_2025_483_MOESM10_ESM.zip › EV2 source data/EV2D/FIP200p62DKO-p62SE-KEAP1_p62.jpg]

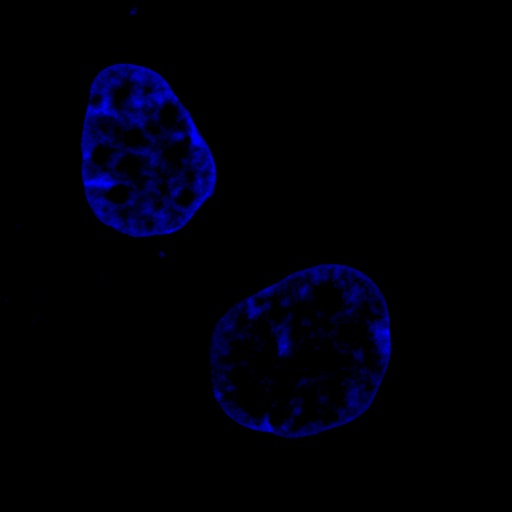

Supplement: Supplementary file 10 — Figure EV2 Source Data [file 44319_2025_483_MOESM10_ESM.zip › EV2 source data/EV2D/FIP200p62DKO-empty vector-KEAP1_DAPI.jpg]

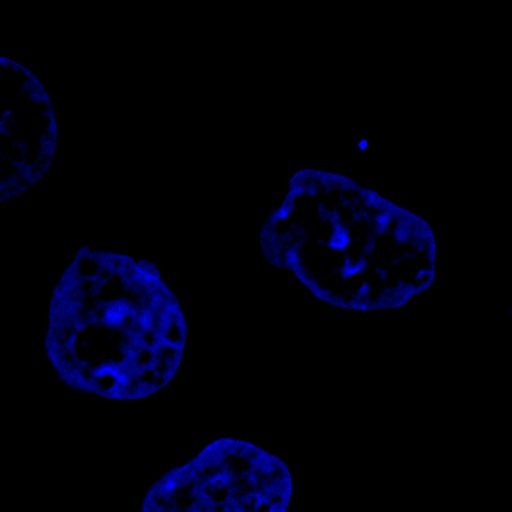

Supplement: Supplementary file 10 — Figure EV2 Source Data [file 44319_2025_483_MOESM10_ESM.zip › EV2 source data/EV2D/FIP200p62DKO-p62SE-KEAP1_DAPI.jpg]

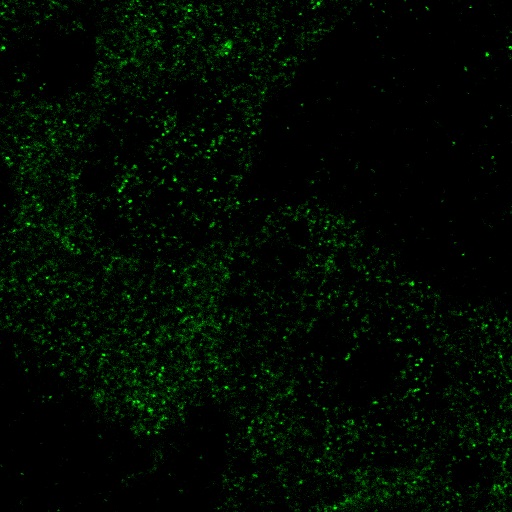

Supplement: Supplementary file 10 — Figure EV2 Source Data [file 44319_2025_483_MOESM10_ESM.zip › EV2 source data/EV2D/FIP200p62DKO-p62TA-KEAP1_KEAP1.jpg]

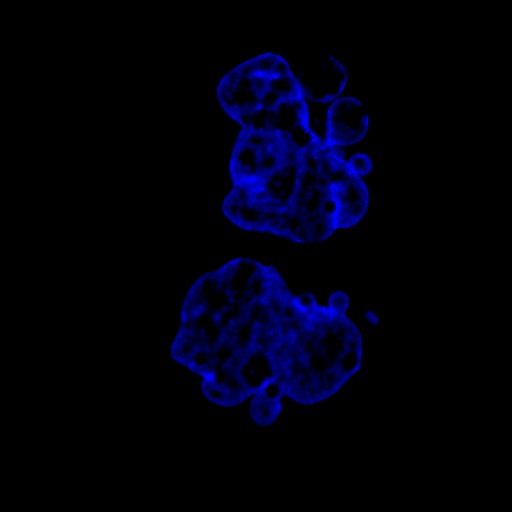

Supplement: Supplementary file 10 — Figure EV2 Source Data [file 44319_2025_483_MOESM10_ESM.zip › EV2 source data/EV2D/FIP200p62DKO-p62wt-KEAP1_DAPI.jpg]

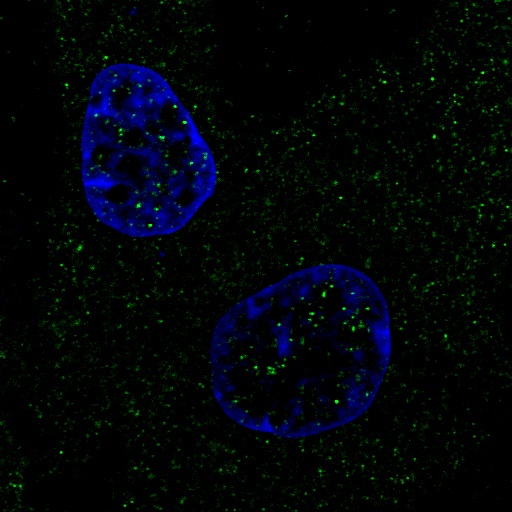

Supplement: Supplementary file 10 — Figure EV2 Source Data [file 44319_2025_483_MOESM10_ESM.zip › EV2 source data/EV2D/FIP200p62DKO-empty vector-KEAP1_Merged.jpg]

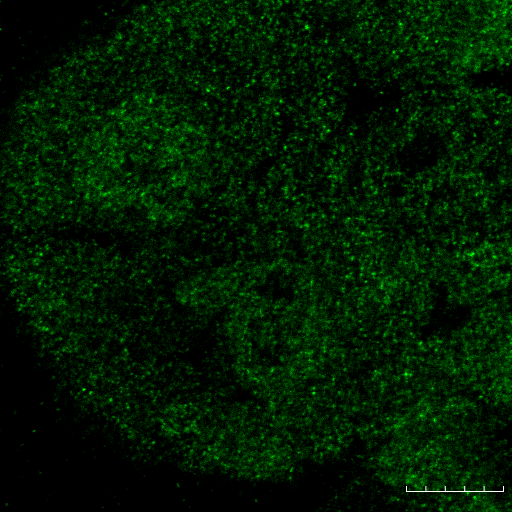

Supplement: Supplementary file 10 — Figure EV2 Source Data [file 44319_2025_483_MOESM10_ESM.zip › EV2 source data/EV2C/p62KO-TA-KEAP1_KEAP1.tif]

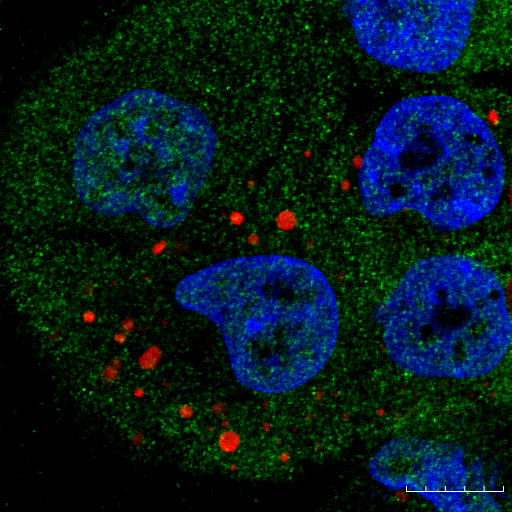

Supplement: Supplementary file 10 — Figure EV2 Source Data [file 44319_2025_483_MOESM10_ESM.zip › EV2 source data/EV2C/p62KO-TA-KEAP1_Merged.tif]

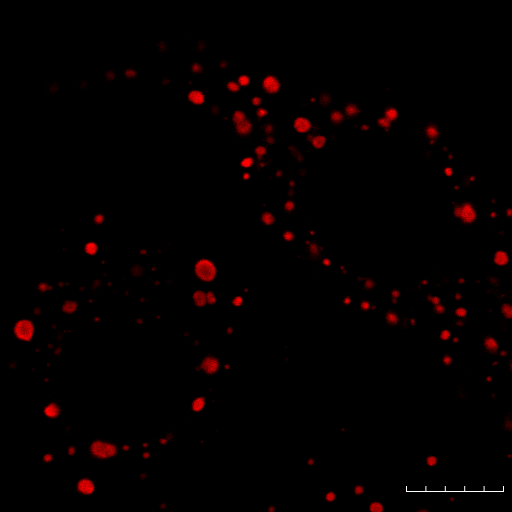

Supplement: Supplementary file 10 — Figure EV2 Source Data [file 44319_2025_483_MOESM10_ESM.zip › EV2 source data/EV2C/p62KO-p62wt-KEAP1_p62.tif]

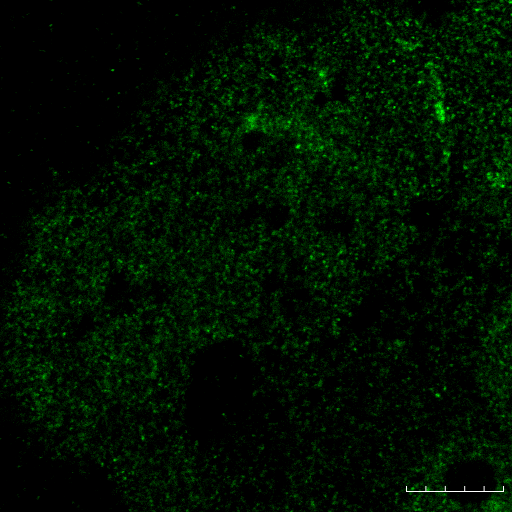

Supplement: Supplementary file 10 — Figure EV2 Source Data [file 44319_2025_483_MOESM10_ESM.zip › EV2 source data/EV2C/p62KO-empty vector-KEAP1_KEAP1.tif]

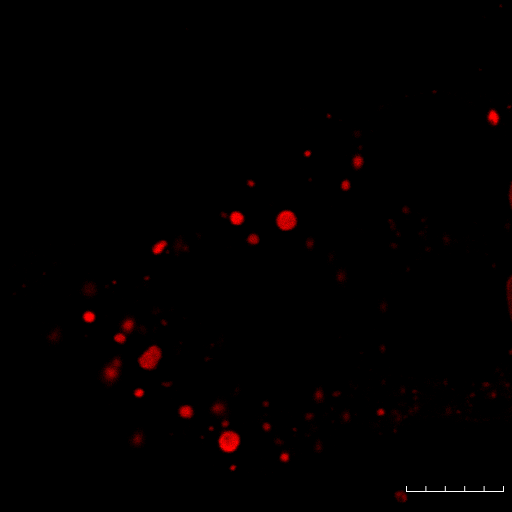

Supplement: Supplementary file 10 — Figure EV2 Source Data [file 44319_2025_483_MOESM10_ESM.zip › EV2 source data/EV2C/p62KO-TA-KEAP1_p62.tif]

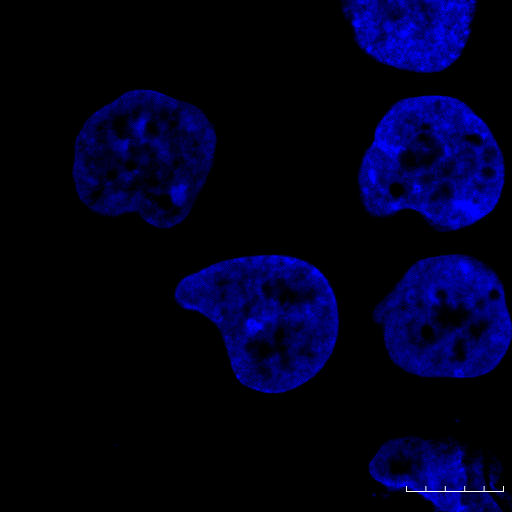

Supplement: Supplementary file 10 — Figure EV2 Source Data [file 44319_2025_483_MOESM10_ESM.zip › EV2 source data/EV2C/p62KO-TA-KEAP1_DAPI.tif]

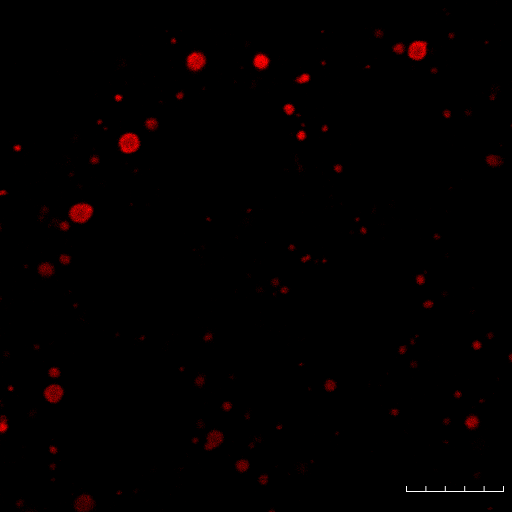

Supplement: Supplementary file 10 — Figure EV2 Source Data [file 44319_2025_483_MOESM10_ESM.zip › EV2 source data/EV2C/p62KO-SA-KEAP1_p62.tif]

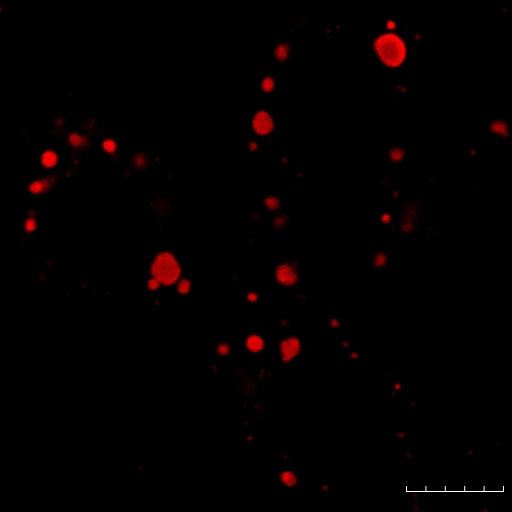

Supplement: Supplementary file 10 — Figure EV2 Source Data [file 44319_2025_483_MOESM10_ESM.zip › EV2 source data/EV2C/p62KO-SE-KEAP1_p62.tif]

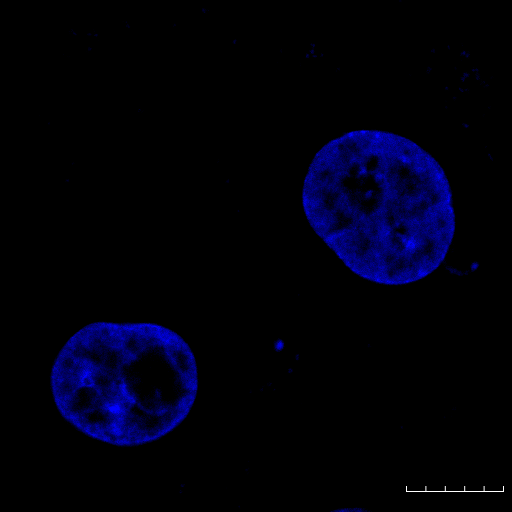

Supplement: Supplementary file 10 — Figure EV2 Source Data [file 44319_2025_483_MOESM10_ESM.zip › EV2 source data/EV2C/p62KO-p62wt-KEAP1_DAPI.tif]

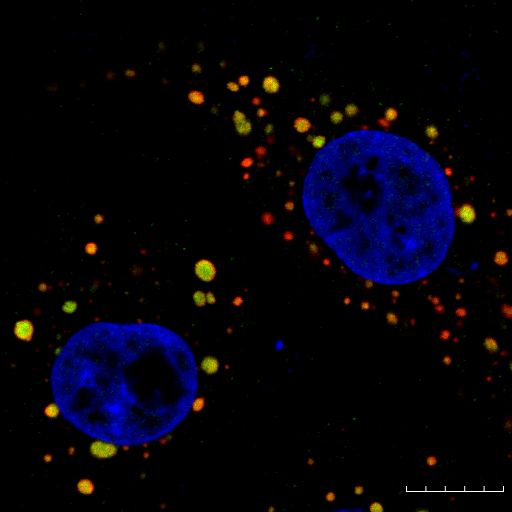

Supplement: Supplementary file 10 — Figure EV2 Source Data [file 44319_2025_483_MOESM10_ESM.zip › EV2 source data/EV2C/p62KO-p62wt-KEAP1_Merged.tif]

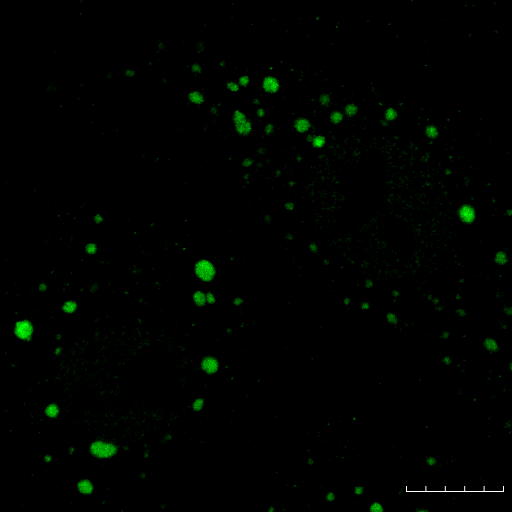

Supplement: Supplementary file 10 — Figure EV2 Source Data [file 44319_2025_483_MOESM10_ESM.zip › EV2 source data/EV2C/p62KO-p62wt-KEAP1_KEAP1.tif]

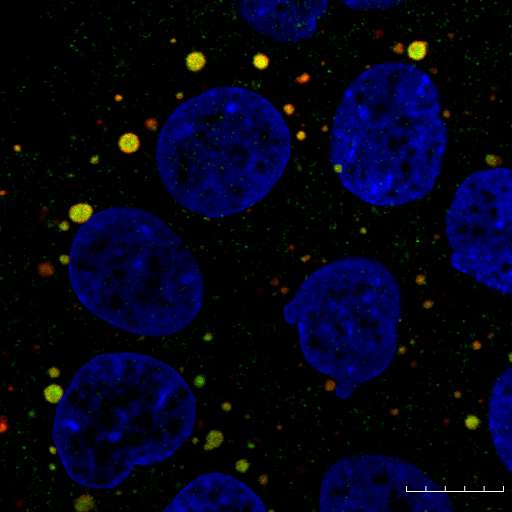

Supplement: Supplementary file 10 — Figure EV2 Source Data [file 44319_2025_483_MOESM10_ESM.zip › EV2 source data/EV2C/p62KO-SA-KEAP1_Merged.tif]

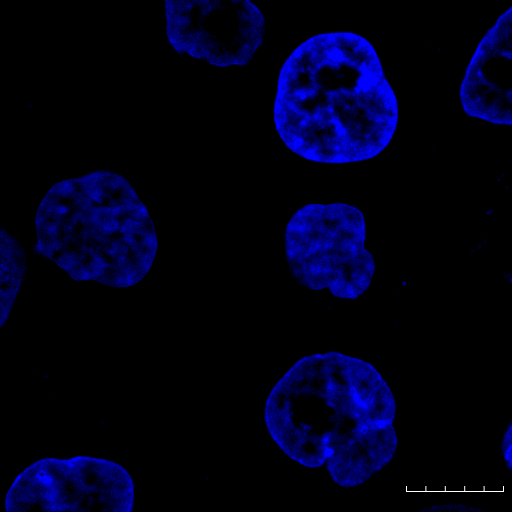

Supplement: Supplementary file 10 — Figure EV2 Source Data [file 44319_2025_483_MOESM10_ESM.zip › EV2 source data/EV2C/p62KO-SE-KEAP1_DAPI.tif]

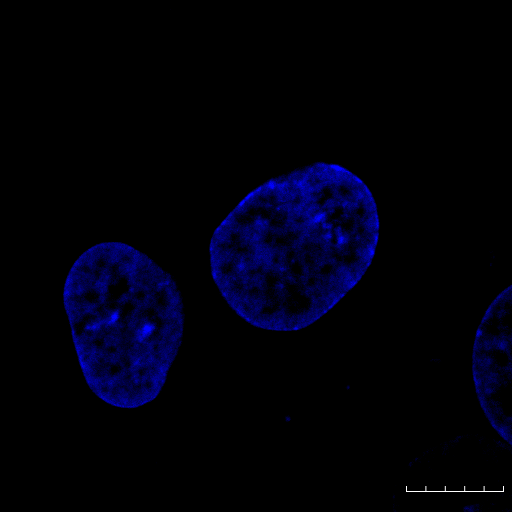

Supplement: Supplementary file 10 — Figure EV2 Source Data [file 44319_2025_483_MOESM10_ESM.zip › EV2 source data/EV2C/p62KO-empty vector-KEAP1_DAPI.tif]

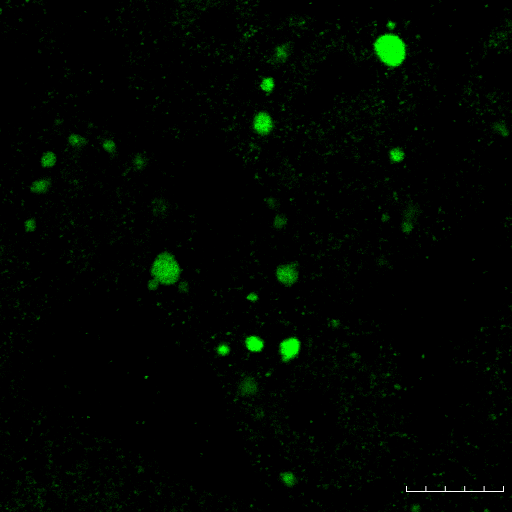

Supplement: Supplementary file 10 — Figure EV2 Source Data [file 44319_2025_483_MOESM10_ESM.zip › EV2 source data/EV2C/p62KO-SE-KEAP1_KEAP1.tif]

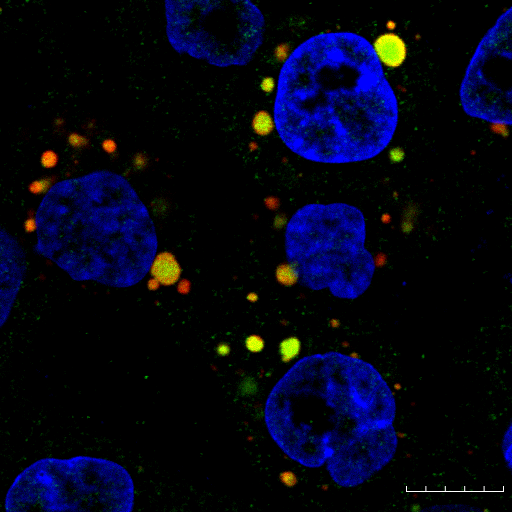

Supplement: Supplementary file 10 — Figure EV2 Source Data [file 44319_2025_483_MOESM10_ESM.zip › EV2 source data/EV2C/p62KO-SE-KEAP1_Merged.tif]

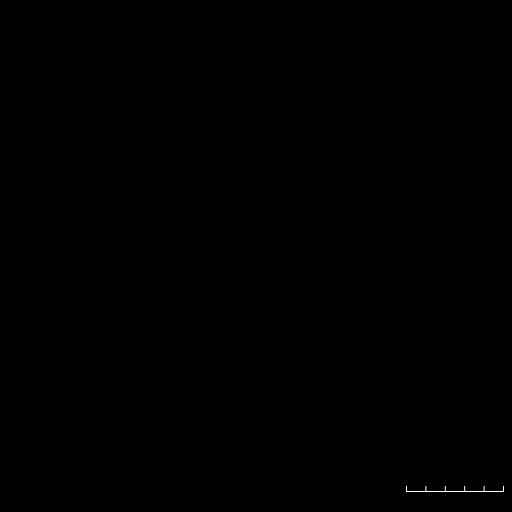

Supplement: Supplementary file 10 — Figure EV2 Source Data [file 44319_2025_483_MOESM10_ESM.zip › EV2 source data/EV2C/p62KO-empty vector-KEAP1_p62.tif]

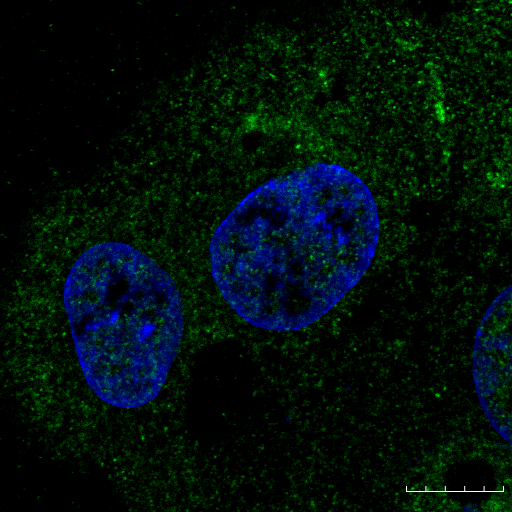

Supplement: Supplementary file 10 — Figure EV2 Source Data [file 44319_2025_483_MOESM10_ESM.zip › EV2 source data/EV2C/p62KO-empty vector-KEAP1_Merged.tif]

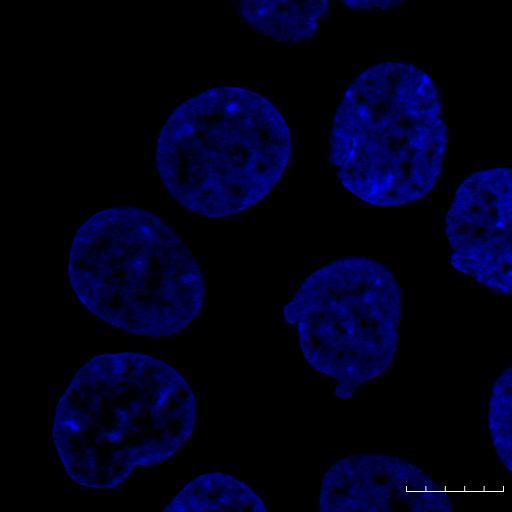

Supplement: Supplementary file 10 — Figure EV2 Source Data [file 44319_2025_483_MOESM10_ESM.zip › EV2 source data/EV2C/p62KO-SA-KEAP_DAPI.tif]

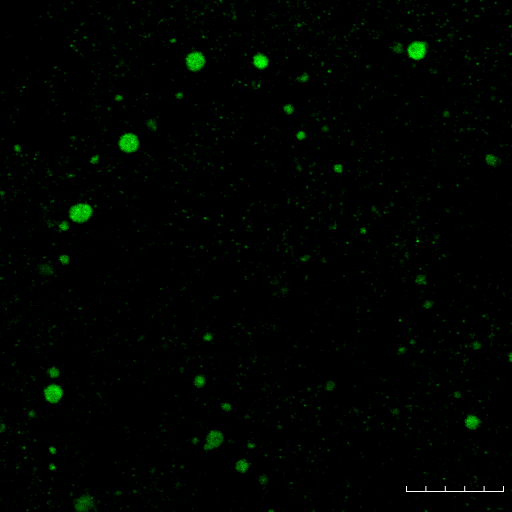

Supplement: Supplementary file 10 — Figure EV2 Source Data [file 44319_2025_483_MOESM10_ESM.zip › EV2 source data/EV2C/p62KO-SA-KEAP1_KEAP1.tif]
