# Supplementary figures and images for: KEAP1 retention in phase-separated p62 bodies drives liver damage under autophagy-deficient conditions (part 2 of 2)
Source: EMBO Rep. 2025 May 28;26(13):3384–410. doi: 10.1038/s44319-025-00483-9 (PMC12238652; doi:10.1038/s44319-025-00483-9)

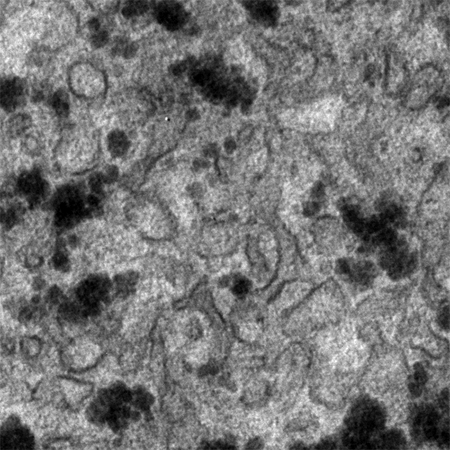

Supplement: Supplementary file 12 — Figure EV4 Source Data [file 44319_2025_483_MOESM12_ESM.zip › EV4 source data/EV4_Atg7_p62T352A inset.tif]

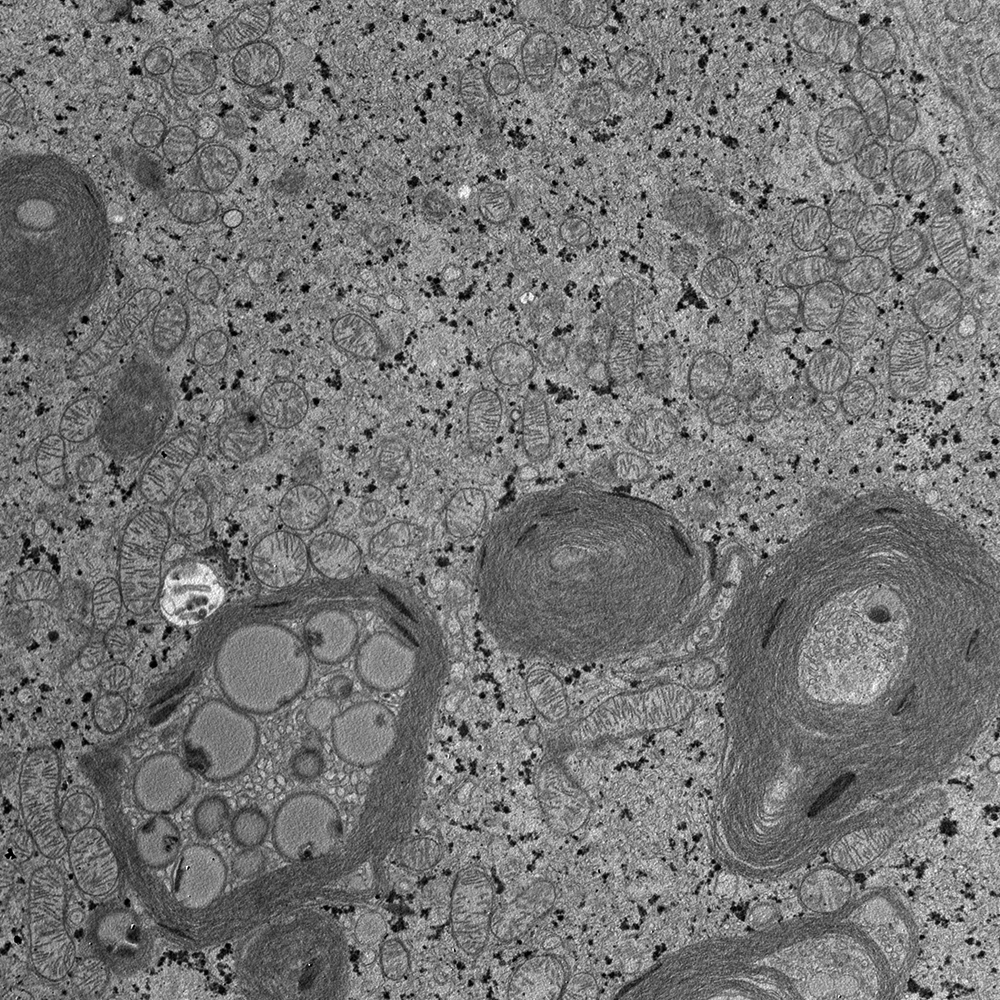

Supplement: Supplementary file 12 — Figure EV4 Source Data [file 44319_2025_483_MOESM12_ESM.zip › EV4 source data/EV4_Atg7 KO.tif]

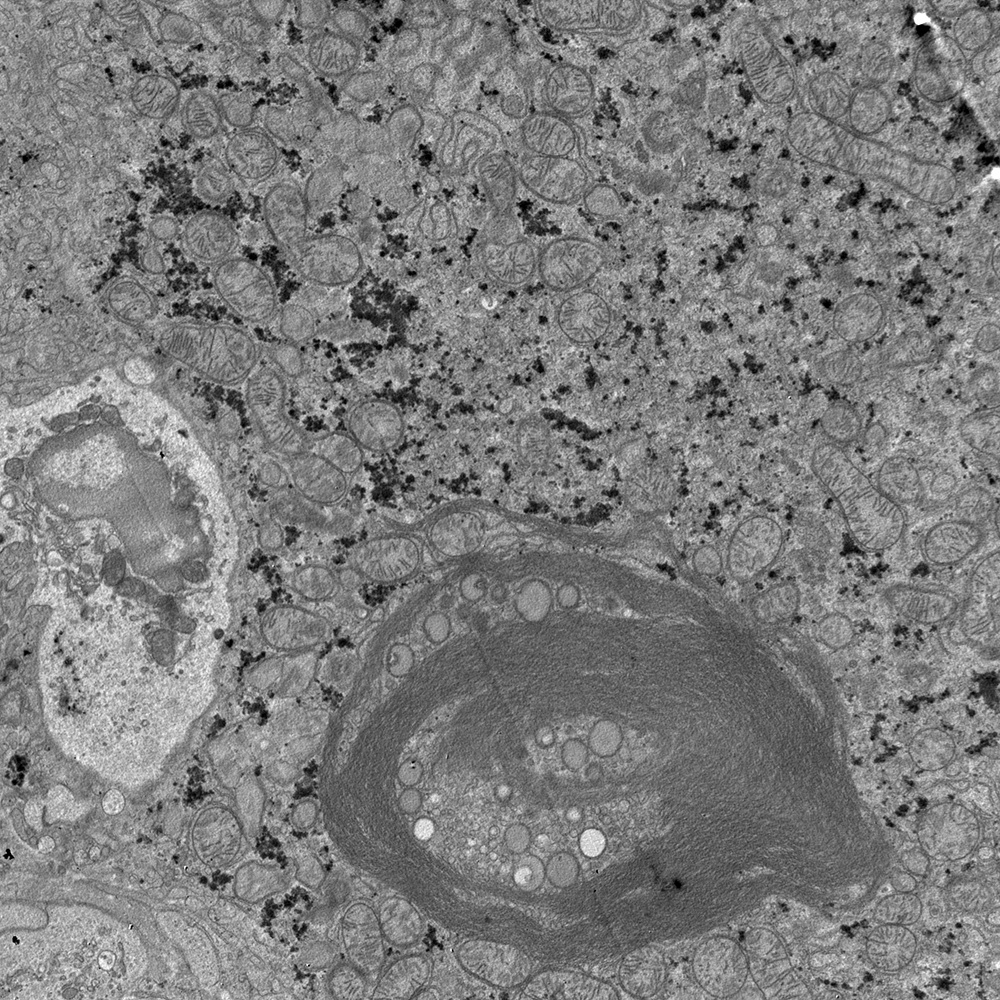

Supplement: Supplementary file 12 — Figure EV4 Source Data [file 44319_2025_483_MOESM12_ESM.zip › EV4 source data/EV4_Atg7_p62T352A.tif]

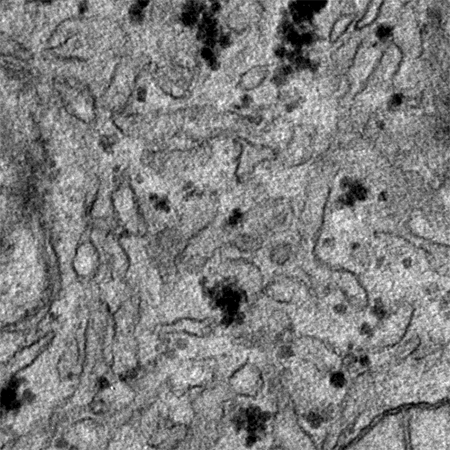

Supplement: Supplementary file 12 — Figure EV4 Source Data [file 44319_2025_483_MOESM12_ESM.zip › EV4 source data/EV4_Atg7 KO inset.tif]

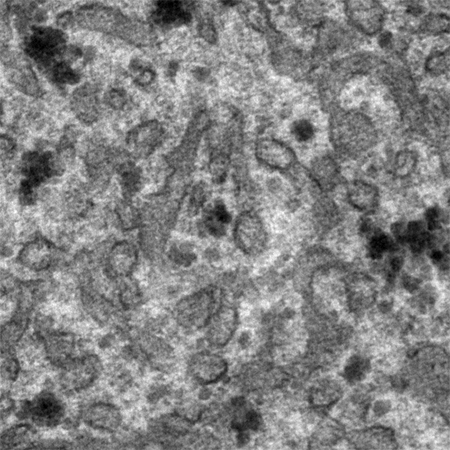

Supplement: Supplementary file 12 — Figure EV4 Source Data [file 44319_2025_483_MOESM12_ESM.zip › EV4 source data/EV4_Atg7_p62S351A inset.tif]

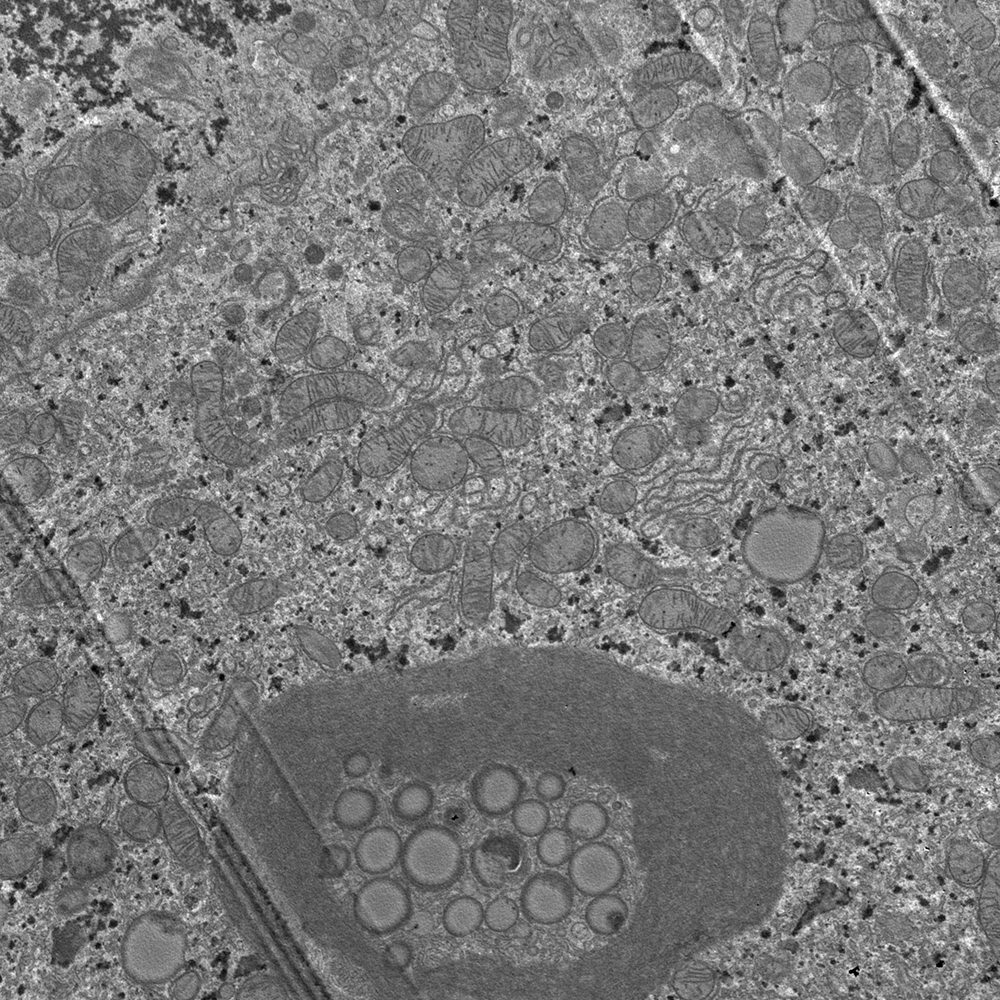

Supplement: Supplementary file 12 — Figure EV4 Source Data [file 44319_2025_483_MOESM12_ESM.zip › EV4 source data/EV4_Atg7_p62S351A.tif]
