# Supplementary material for: KEAP1 retention in phase-separated p62 bodies drives liver damage under autophagy-deficient conditions
Source: EMBO Rep. 2025 May 28;26(13):3384–410. doi: 10.1038/s44319-025-00483-9 (PMC12238652; doi:10.1038/s44319-025-00483-9)
Supplement: Supplementary file 13 — Expanded View Figures [file 44319_2025_483_MOESM13_ESM.pdf]

Expanded View Figures

Figure EV1. Accumulation of p62 bodies in *Atg7*-deficient hepatocytes.

(**A**) Immunofluorescence microscopy. Primary cultured hepatocytes isolated from 5-week-old *Atg7*$^{flox/flox}$ and *Atg7*$^{flox/flox}$;Alb-*Cre* mice were immunostained with anti-p62 antibody. Scale bars: 10 µm. (**B**) Electron microscopy. Representative electron micrographs of cytoplasmic regions in primary cultured hepatocytes from 5-week-old *Atg7*$^{flox/flox}$;Alb-*Cre* mice. Boxed region is displayed at higher magnification. Typical p62 bodies, along with concentric membranous structures associated with the endoplasmic reticulum (ER), accumulated in *Atg7*-deficient hepatocytes. Arrow indicates the concentric membranous structure, while arrowheads indicate p62 bodies. Scale bars: 5 µm; 500 nm. (**C**) Immunoblot analysis. Wild-type ATG7 or the active-site mutant ATG7^{C572S} was introduced into *Atg7*-knockout primary cultured hepatocytes (isolated from 5-week-old *Atg7*$^{flox/flox}$;Alb-*Cre* mice) using an adenoviral system. Cell lysates were collected at the indicated time points after infection and subjected to immunoblot analysis with the specified antibodies. The bar graphs present the quantitative densitometric analysis of p62, Ser351-phosphorylated p62, and KEAP1, normalized to GAPDH ($n = 3$). Data are presented as means ± s.e. Statistical analysis was performed using a one-way ANOVA followed by Tukey's test. (**D**) Immunofluorescence microscopy. Wild-type ATG7 or the active-site mutant ATG7^{C572S} was introduced into *Atg7*-knockout primary cultured hepatocytes (isolated from 5-week-old *Atg7*$^{flox/flox}$;Alb-*Cre* mice) using an adenoviral system. Immunostaining was performed at the indicated time points after infection with a p62 antibody. The size and number of p62 bodies per cell were quantified ($n = 500$ cells). Horizontal bars indicate medians, boxes represent the interquartile range (25th–75th percentiles), and whiskers extend to 1.5× the interquartile range; individual outliers are shown as points. Statistical analysis was performed using Welch's *t* test. Scale bars: 10 µm (main panels), 1 µm (inset panels). Scale bars: 10 µm. Source data are available online for this figure.

© The Author(s)

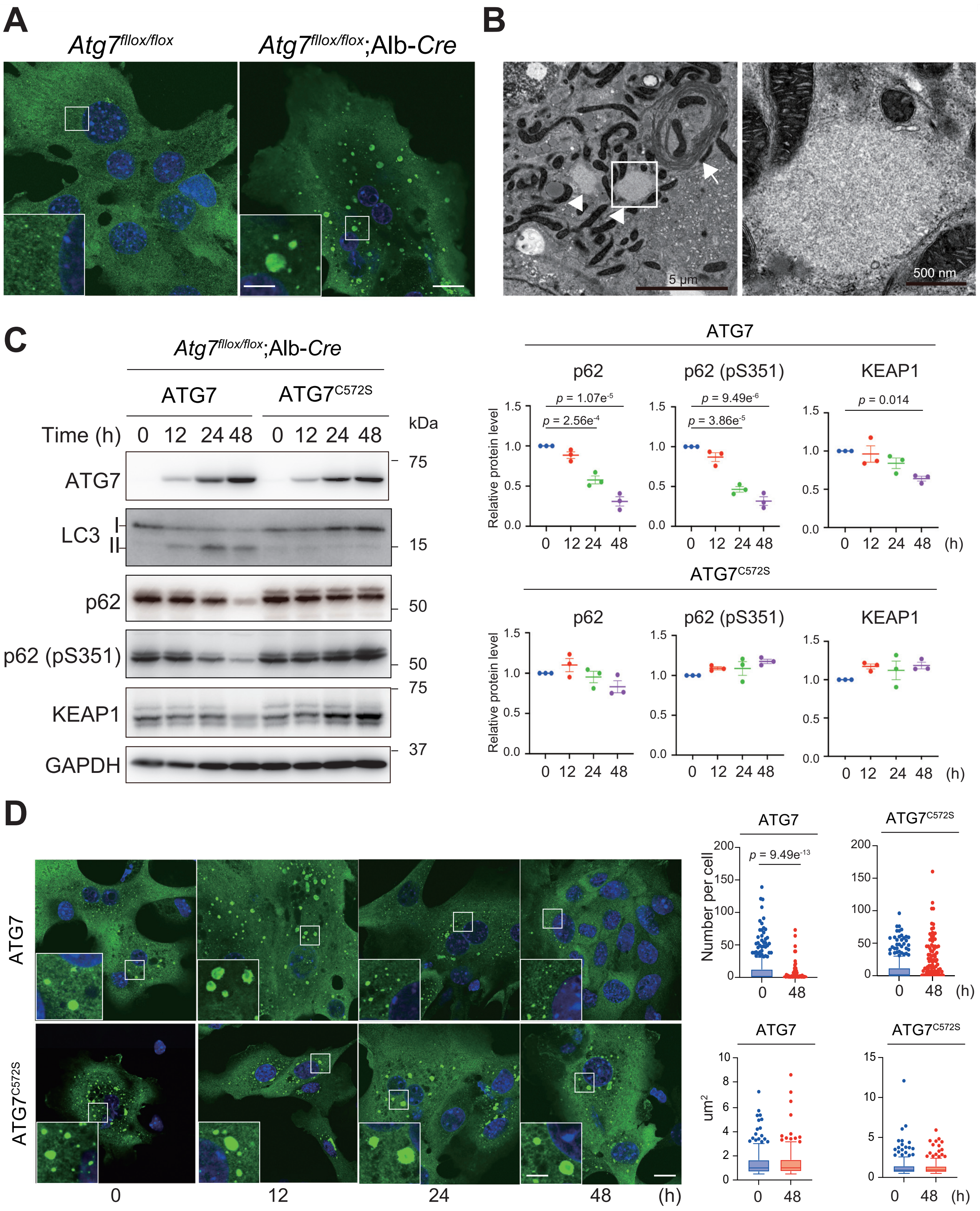
A
Atg7flox/flox
Atg7flox/flox;Alb-Cre
B
5 μm
500 nm
C
Atg7flox/flox;Alb-Cre
ATG7
ATG7C572S
Time (h) 0 12 24 48 0 12 24 48
kDa
ATG7
75
LC3 I II
15
p62
50
p62 (pS351)
50
75
KEAP1
37
GAPDH
ATG7
p62
p62 (pS351)
KEAP1
p = 1.07e-5
p = 2.56e-4
p = 9.49e-6
p = 3.86e-5
p = 0.014
Relative protein level
0 12 24 48 (h)
ATG7C572S
D
ATG7
ATG7C572S
0 12 24 48 (h)
Number per cell
p = 9.49e-13
um2

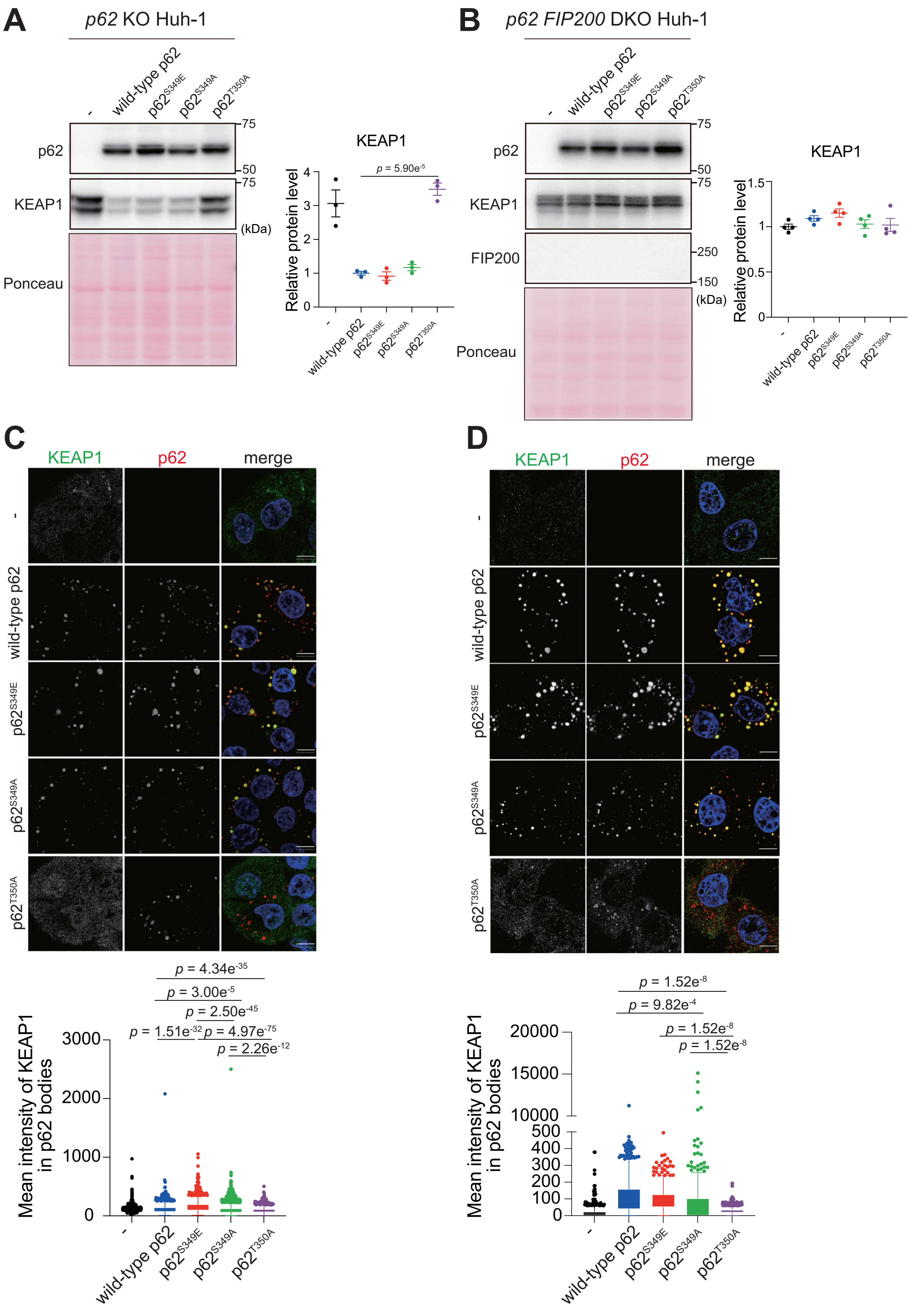
A
p62 KO Huh-1
-
wild-type p62
p62S349E
p62S349A
p62T350A
p62
KEAP1
Ponceau
75
50
75
(kDa)
KEAP1
p = 5.90e-5
Relative protein level
B
p62 FIP200 DKO Huh-1
FIP200
250
150
C
KEAP1
p62
merge
D
p = 4.34e-35
p = 3.00e-5
p = 2.50e-45
p = 1.51e-32
p = 4.97e-75
p = 2.26e-12
Mean intensity of KEAP1 in p62 bodies
p = 1.52e-8
p = 9.82e-4
p = 1.52e-8
p = 1.52e-8

◀ **Figure EV2. KEAP1 protein level in cells expressing p62 mutants.**

(**A**, **B**) Immunoblot analysis. Wild-type p62 and indicated p62 mutants were introduced into *p62*-knockout (**A**) or *p62* and *FIP200*-double knockout (**B**) Huh-1 cells, and the cell lysates were subjected to immunoblot analysis with indicated antibodies. Bar graph shows the results of quantitative densitometric analysis of KEAP1 relative to the whole protein content estimated using Ponceau-S staining ($n = 3$). Data are means ± s.e. Statistical analysis was performed by Tukey test after one-way ANOVA. (**C**) Immunofluorescence microscopy. Huh-1 cells indicated in (**A**) were immunostained with the indicated antibodies. Scale bars, 10 µm (main panels). The graph shows the mean intensity of KEAP1 in p62 bodies comprising of wild-type p62 ($n = 2469$), $p62^{S349E}$ ($n = 1057$), $p62^{S349A}$ ($n = 2992$), or $p62^{T350A}$ ($n = 2033$). Data are means ± s.e. Statistical analysis was performed by Tukey test after one-way ANOVA. (**D**) Immunofluorescence microscopy. Huh-1 cells indicated in (**B**) were immunostained with the indicated antibodies. Scale bars, 10 µm (main panels). The graph shows the mean intensity of KEAP1 in p62 bodies comprising of wild-type p62 ($n = 2892$), $p62^{S349E}$ ($n = 2611$), $p62^{S349A}$ ($n = 5191$), or $p62^{T350A}$ ($n = 3522$). Data are means ± s.e. Statistical analysis was performed by Tukey test after one-way ANOVA. Source data are available online for this figure.

© The Author(s)

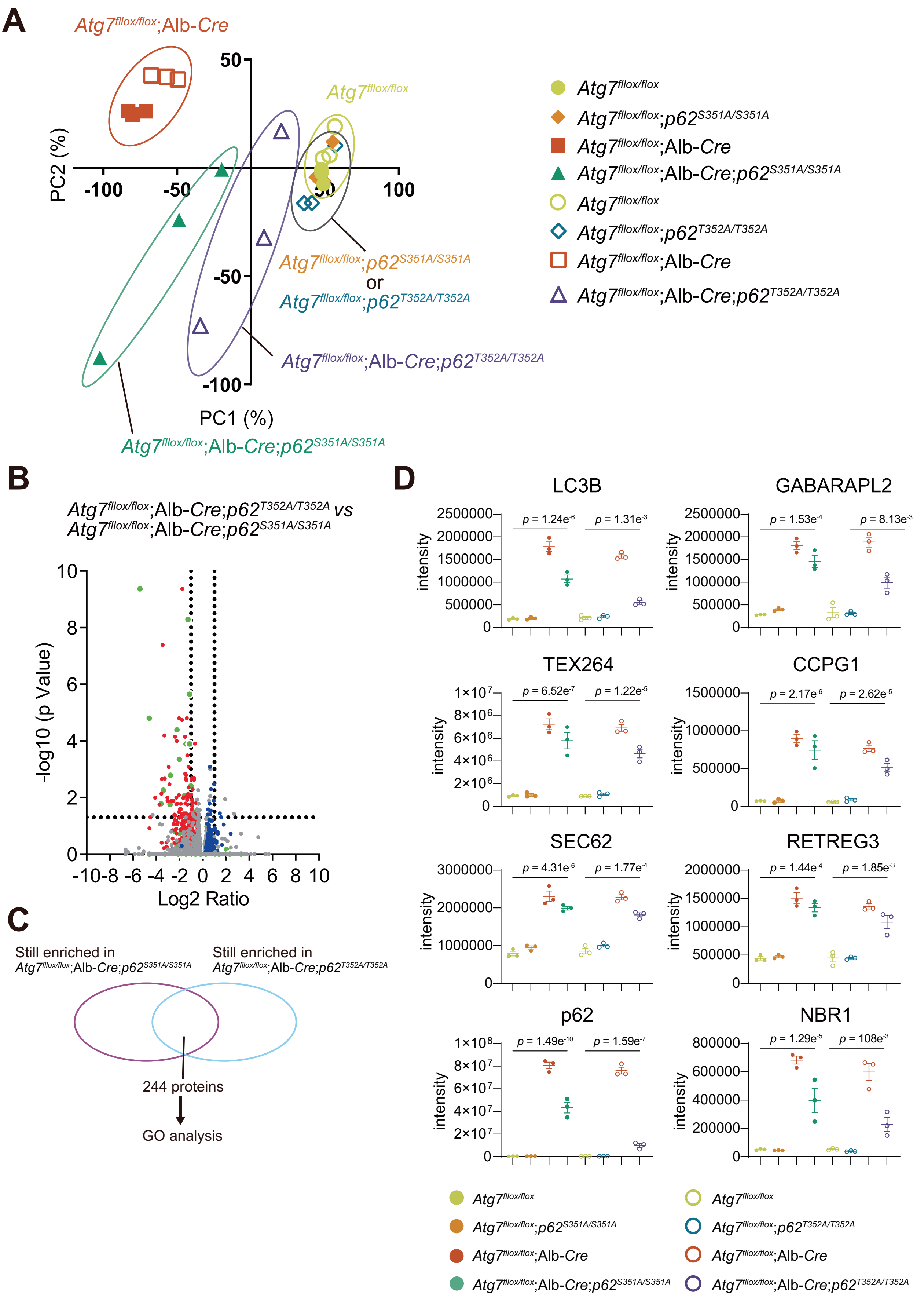
A
Atg7fllox/flox;Alb-Cre
Atg7fllox/flox
Atg7fllox/flox;p62S351A/S351A
or
Atg7fllox/flox;p62T352A/T352A
Atg7fllox/flox;Alb-Cre;p62T352A/T352A
Atg7fllox/flox;Alb-Cre;p62S351A/S351A
PC2 (%)
PC1 (%)
50
-50
-100
-100
-50
100
Atg7fllox/flox
Atg7fllox/flox;p62S351A/S351A
Atg7fllox/flox;Alb-Cre
Atg7fllox/flox;Alb-Cre;p62S351A/S351A
Atg7fllox/flox
Atg7fllox/flox;p62T352A/T352A
Atg7fllox/flox;Alb-Cre
Atg7fllox/flox;Alb-Cre;p62T352A/T352A
B
Atg7fllox/flox;Alb-Cre;p62T352A/T352A vs
Atg7fllox/flox;Alb-Cre;p62S351A/S351A
-log10 (p Value)
Log2 Ratio
-10-8 -6 -4 -2 0 2 4 6 8 10
C
Still enriched in
Atg7fllox/flox;Alb-Cre;p62S351A/S351A
Still enriched in
Atg7fllox/flox;Alb-Cre;p62T352A/T352A
244 proteins
GO analysis
D
LC3B
p = 1.24e-6
p = 1.31e-3
GABARAPL2
p = 1.53e-4
p = 8.13e-3
TEX264
p = 6.52e-7
p = 1.22e-5
CCPG1
p = 2.17e-6
p = 2.62e-5
SEC62
p = 4.31e-6
p = 1.77e-4
RETREG3
p = 1.44e-4
p = 1.85e-3
p62
p = 1.49e-10
p = 1.59e-7
NBR1
p = 1.29e-5
p = 108e-3
intensity
Atg7fllox/flox
Atg7fllox/flox;p62S351A/S351A
Atg7fllox/flox;Alb-Cre
Atg7fllox/flox;Alb-Cre;p62S351A/S351A
Atg7fllox/flox
Atg7fllox/flox;p62T352A/T352A
Atg7fllox/flox;Alb-Cre
Atg7fllox/flox;Alb-Cre;p62T352A/T352A

Figure EV3. Proteomic analysis of hepatocyte-specific *Atg7*-knockout mice with different p62 mutations.

(**A**) Principal component analysis (PCA) of mouse liver proteomic data based on triplicate biological replicates. Samples of the same genotype are circled. (**B**) Proteomic comparison of *Atg7*-KO;*p62*^{S351A} and *Atg7*-KO;*p62*^{T352A}. Proteins accumulated as shown in Fig. 5B are indicated in red, and those with decreased abundance are shown in blue. NRF2 target genes are marked in green. (**C**) Venn diagram of proteomic data showing proteins that accumulated more than 2-fold in both *Atg7*-KO;*p62*^{S351A} and *Atg7*-KO;*p62*^{T352A} mouse livers compared to controls. Gene Ontology (GO) analysis was performed on 244 proteins common to both genotypes. (**D**) Label-free quantification of autophagy-related and selective autophagy receptor proteins based on MS intensity. The bar graph shows quantitative intensity of autophagy proteins normalized to the total peptide amount, with unadjusted *P* values from one-way ANOVA for individual proteins ($n = 3$). Data are means ± s.d. Differential protein expressions were assessed using one-way ANOVA, with *P* values adjusted for multiple comparisons using the Benjamini–Hochberg method. Source data are available online for this figure.

 © The Author(s)

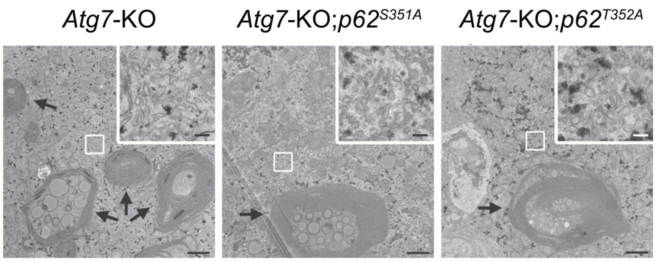

Figure EV4. Electron micrographs of hepatocytes in hepatocyte-specific *Atg7*-knockout mice with different p62 mutations.

Electron micrographs of cytoplasmic regions from hepatocytes of the indicated genotypes. Boxed regions are shown at higher magnification in the insets. Concentric membranous structures connected to the endoplasmic reticulum (ER) are present in hepatocytes of all genotypes (arrows). Numerous ER profiles are also visible (insets). Scale bars: 1 μm and 0.2 μm (insets). Source data are available online for this figure.
